# Supplementary material for: Nuclear depletion of RNA-binding protein ELAVL3 (HuC) in sporadic and familial amyotrophic lateral sclerosis
Source: Acta Neuropathol. 2021 Oct 7;142(6):985–1001. doi: 10.1007/s00401-021-02374-4 (PMC8568872; doi:10.1007/s00401-021-02374-4)
Supplement: Supplementary file 1 — Supplementary file1 (PDF 317349 KB) [file 401_2021_2374_MOESM1_ESM.pdf]

Supplementary data.

Nuclear depletion of RNA binding protein ELAVL3 (HuC) in sporadic and familial amyotrophic lateral sclerosis.

Sandra Diaz-Garcia, PhD[1] sdiazgarcia@health.ucsd.edu
Vivian I. Ko, BS[1] vko@health.ucsd.edu
Sonia Vazquez-Sanchez, PhD[2,3] soniavazquez-sanchez@health.ucsd.edu
Ruth Chia, PhD[4] ruth.chia@nih.gov
Olubankole Aladesuyi Arogundade, PhD[1] oaladesu@health.ucsd.edu
Maria J Rodriguez, BS[1] mjrodriguez@health.ucsd.edu
Don Cleveland, PhD[2,3] dcleveland@health.ucsd.edu
Bryan J. Traynor, MD, PhD[4] traynorb@mail.nih.gov
John Ravits, MD[1] jravits@health.ucsd.edu

[1] Department of Neurosciences, University of California, San Diego, La Jolla, CA 92093-0670
[2] Department of Cellular and Molecular Medicine, University of California, San Diego, La Jolla, CA 92093-0670.
[3] Ludwig Institute for Cancer Research, University of California at San Diego, La Jolla, CA, USA.
[4] Laboratory of Neurogenetics, National Institute on Aging, National Institutes of Health, Bethesda, MD 20892-3707. Department of Neurology, Johns Hopkins University, Baltimore, MD 21287, USA Reta Lila Weston Institute, UCL Queen Square Institute of Neurology, University College London, London, UK. National Institute on Neurological Disorders and Stroke, Bethesda, MD 20892, USA

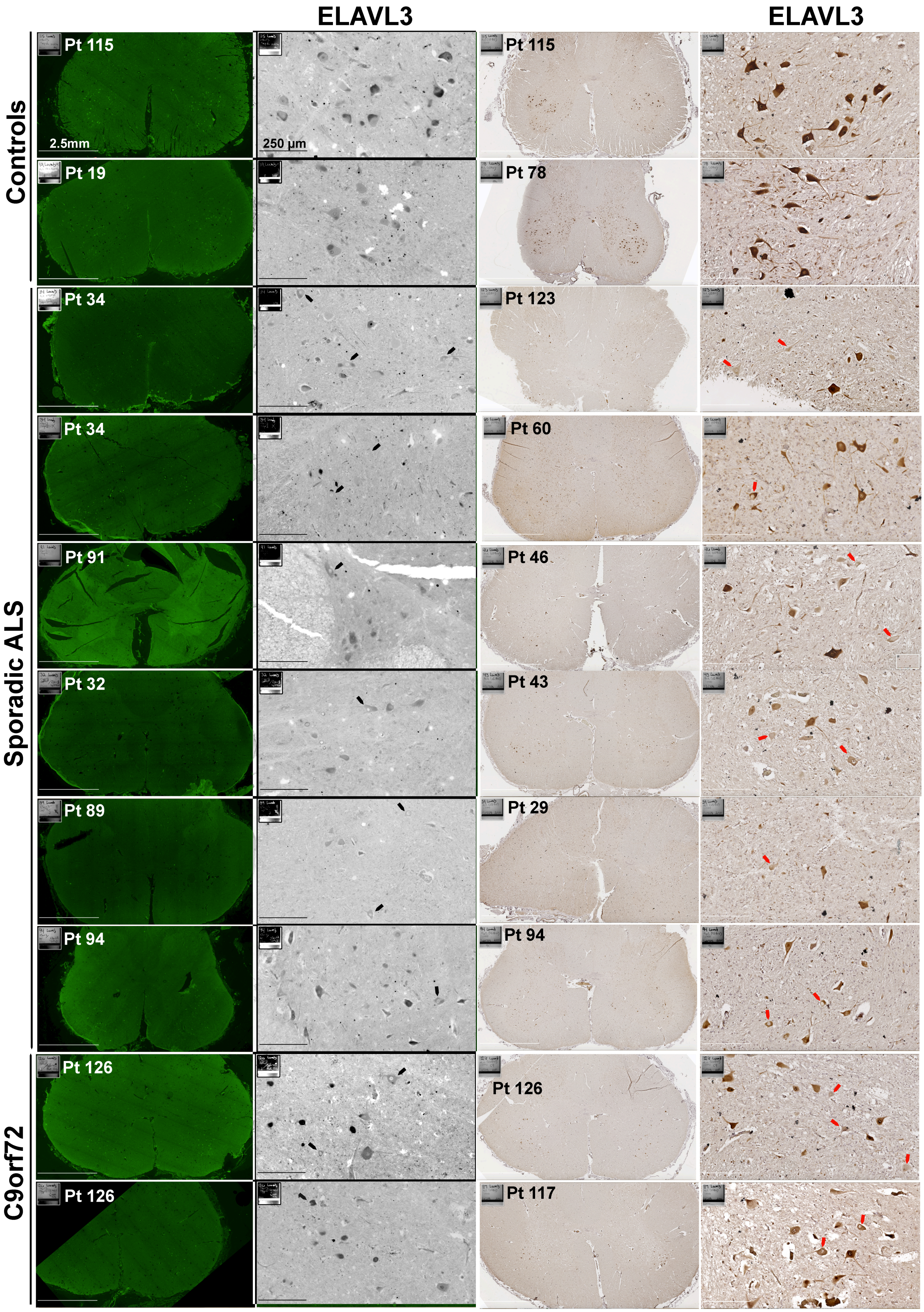

Supplementary Figure 1. ELAVL3 depletion using two different antibodies and imaging techniques Proteintech antibody is used for IF and LsBio is used for IHC.

Supplementary Figure 1. ELAVL3 depletion using two different antibodies and imaging techniques **Proteintech antibody is used for IF and LsBio is used for IHC.**

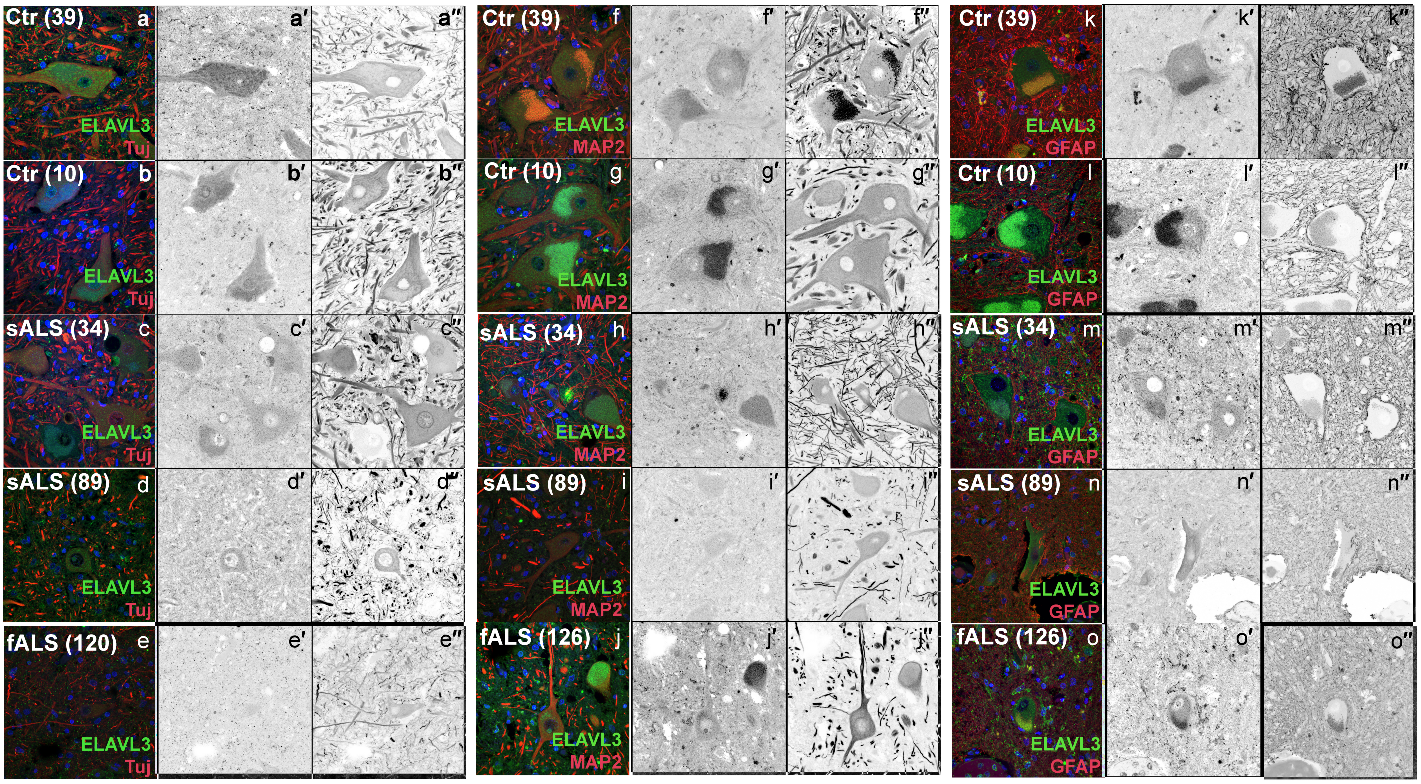

Supplementary Figure 2. ELAVL3 is expressed in the nucleus and cytoplasm of neurons and can also be seen in axons and dendrites.

Supplementary Figure 2. ELAVL3 is expressed in the nucleus and cytoplasm of neurons and can also be seen in axons and dendrites. A-E. Co-staining of ELAVL3 and Tuj1 show no clear accumulation of ELAVL3 in axons in ALS. F-J. Co-staining of ELAVL3 and MAP2 show no clear accumulation of ELAVL3 in dendrites in ALS and rarely in C9orf72. K-O. Co-staining of ELAVL3 and GFAP show no co-localization in ALS.

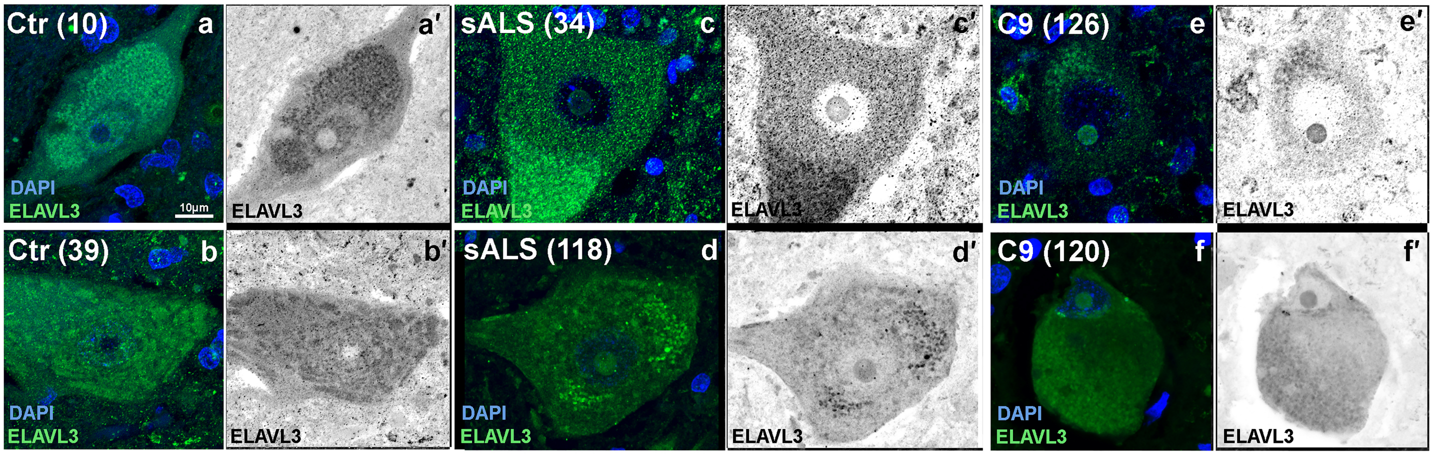

Supplementary Figure 3. ELAVL3 frequently accumulates in nucleolus of ALS but only rarely in control motor neurons.

Supplementary Figure 3. ELAVL3 frequently accumulates in nucleolus of ALS but only rarely in control motor neurons. A-B. Control motor neurons with nuclear ELAVL3 and visible nucleolus without ELAVL3. C-F. ALS motor neurons show nuclear depletion of ELAVL3 and an abnormal accumulation in nucleolus.

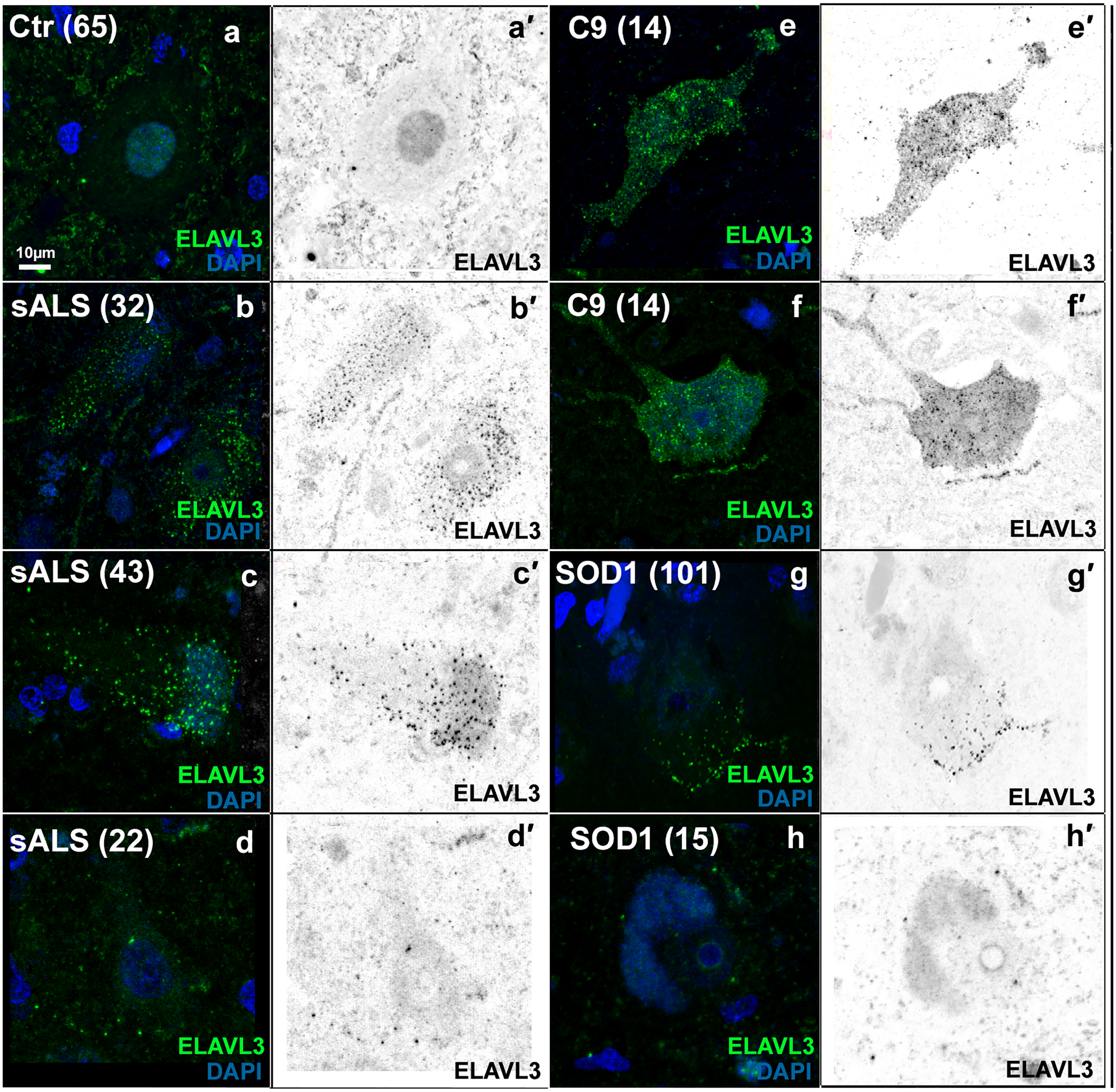

Supplementary Figure 4. ELAVL3 is mislocalized and forms aggregates in motor cortex of ALS patients.

Supplementary Figure 4. ELAVL3 is mislocalized and forms aggregates in motor cortex of ALS patients. A. Control motor neurons where ELAVL3 is nuclear. (B-D): Sporadic ALS patients motor neurons can have nuclear ELAVL3 (lower neuron in panel B) but also nuclear depleted ELAVL3 as well as cytoplasmic inclusions (B-D). (E-F): C9orf72 motor neurons from motor cortex also have nuclear ELAVL3 (F), nuclear clearance (E) and cytoplasmic inclusions (E-F). (G-H): SOD1 motor neurons from motor cortex also can have nuclear depletion of ELAVL3 (G-H), and cytoplasmic inclusions (G).

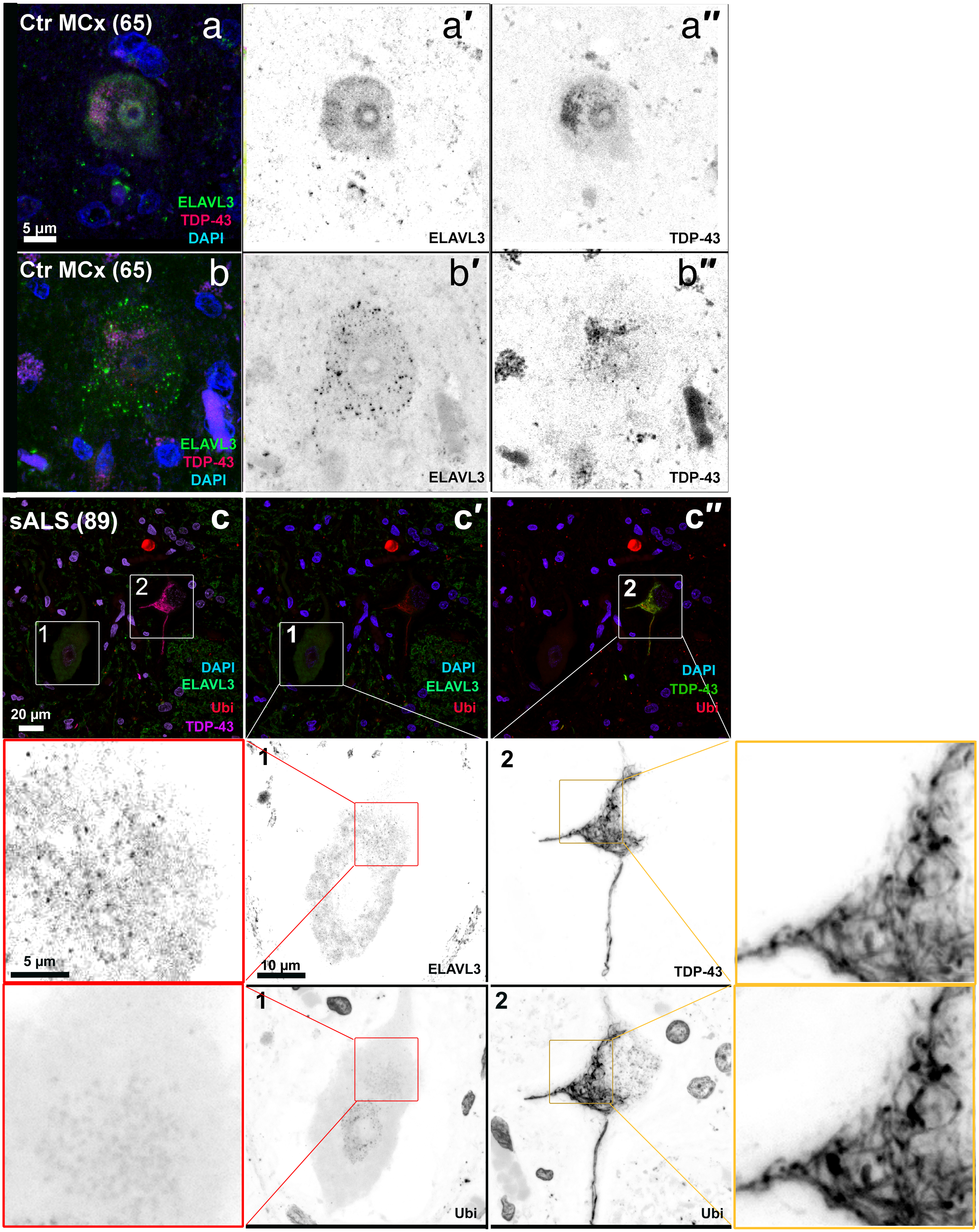

Supplementary Figure 5. Interesting control individuals with rare ELAVL3 abnormalities. and ubiquitination of cytomlasmic inclusions.

Supplementary Figure 5. Interesting control individuals with rare ELAVL3 abnormalities and ubiquitinated cytoplasmic inclusions. (a-b): A few neurons at motor cortex from control 65 that carried intermediate length repeat expansions of ATXN2 had ELAVL3 inclusions in the cytoplasm. (c) Co-stain of ELAVL3, TDP-43 and ubiquitin and magnification of neuron 1 and 2 at the bottom with their respective magnification where cytoplasmic inclusions of ELAVL3 (left) and TDP-43 (right) are present.

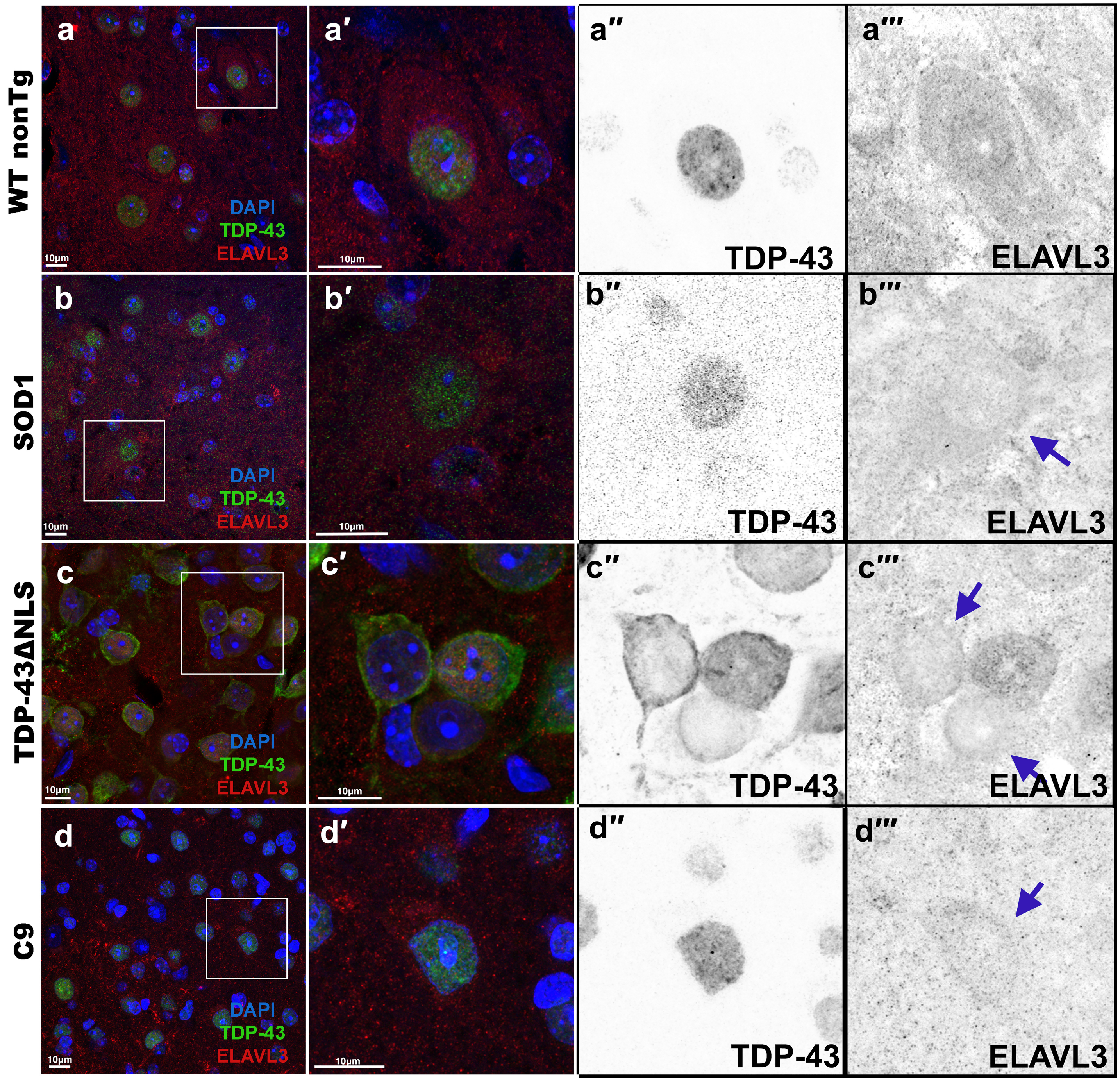

Supplementary Figure 6. ALS mouse models show nuclear depletion of ELAVL3 in motor neurons.

Supplementary Figure 6. ALS mouse models show nuclear depletion of ELAVL3 in motor neurons. (A): Non-transgenic mice show nuclear ELAVL3 and TDP-43 in motor neurons. (B): SOD1 transgenic mutant mice have lack of nuclear ELAVL3 but normal nuclear TDP-43. (C): TDP-43$^{\Delta NLS}$ transgenic mutant mice have lack of both nuclear ELAVL3 and TDP-43 and in addition have cytoplasmic TDP-43 aggregates. (D): C9orf72 mutant mice have lack of nuclear ELAVL3 but have normal nuclear TDP-43. [Scale bar 10μm]

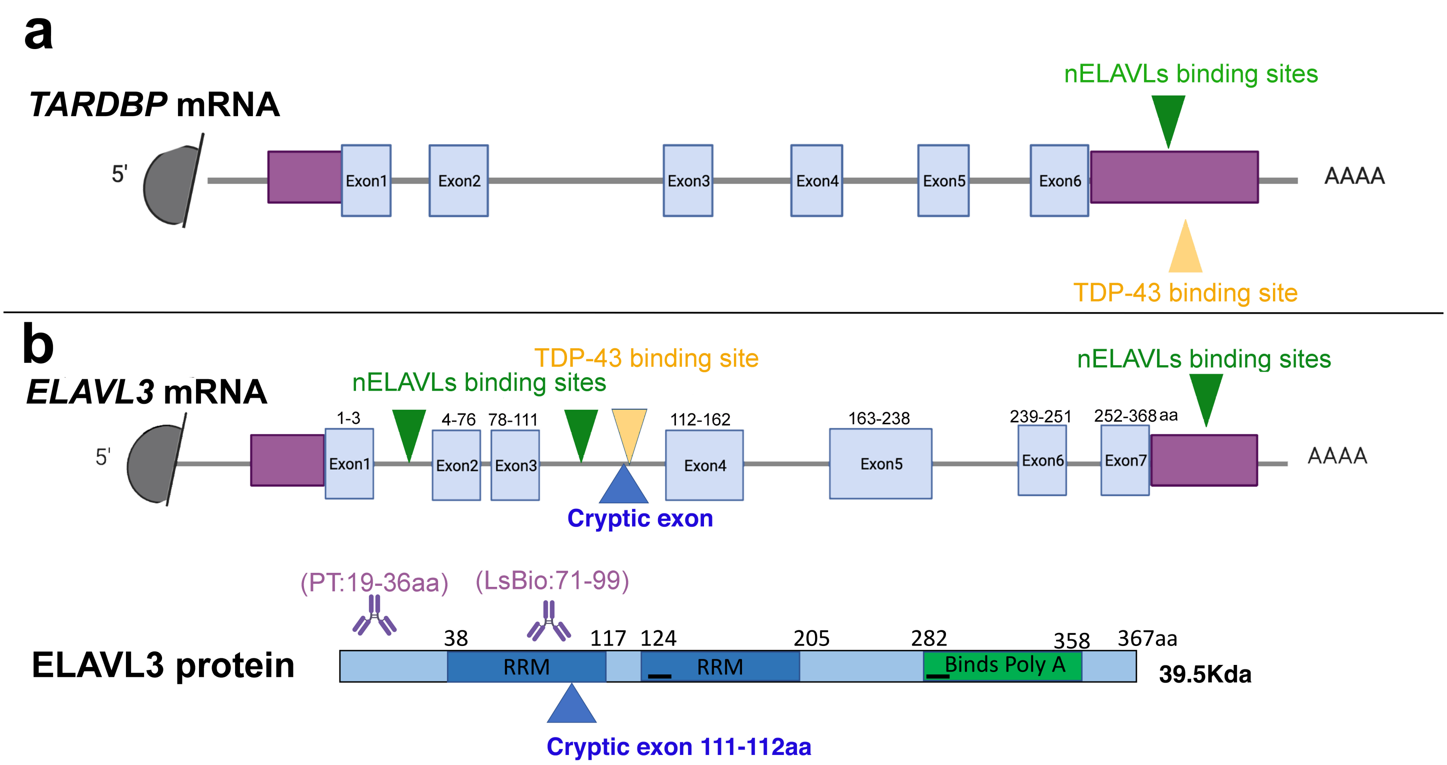

Supplementary Figure 7. No TDP-43-dependent cryptic exon in ELAVL3

Supplementary Figure 7. No TDP-43-dependent cryptic exon in ELAVL3. (A) mRNA binding sites in *TARDBP* by nELAVLs and TDP-43, showing binding is at 3′UTR. (B) mRNA binding sites in *ELAVL3* by nELAVLs and TDP-43, showing binding in introns and location of TDP-43-dependent cryptic exon in intron 3.

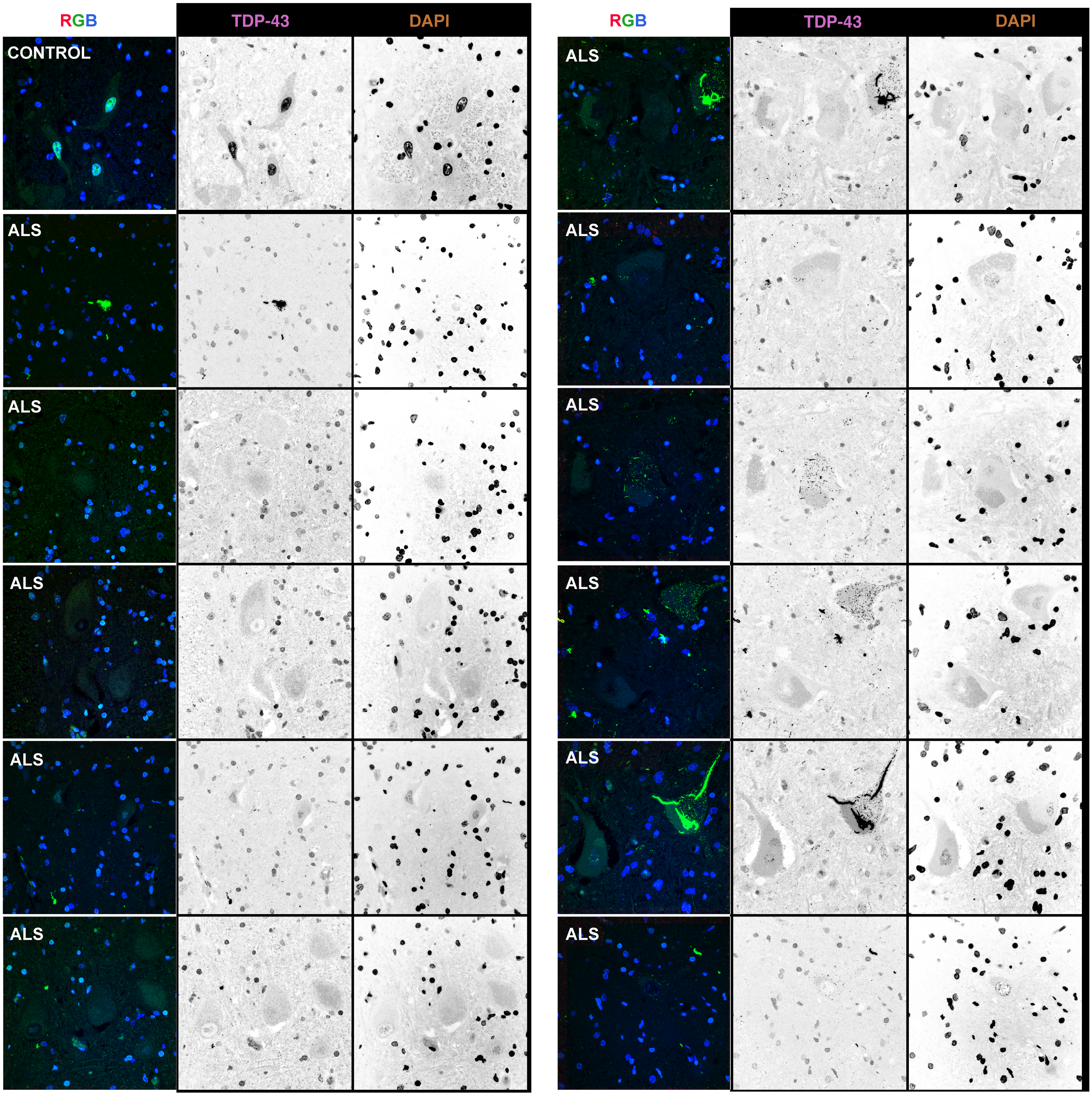

Supplementary Figure 8. Different examples of TDP-43 pathology in neurons and glial cell in controls and ALS motor neurons.

Supplementary Figure 8. Different examples of TDP-43 pathology in neurons and glial cell in controls and ALS motor neurons. Compilation of images of TDP-43 in control and ALS neurons and glial cells showing a variety of expression in the anterior horn of spinal cord sections.

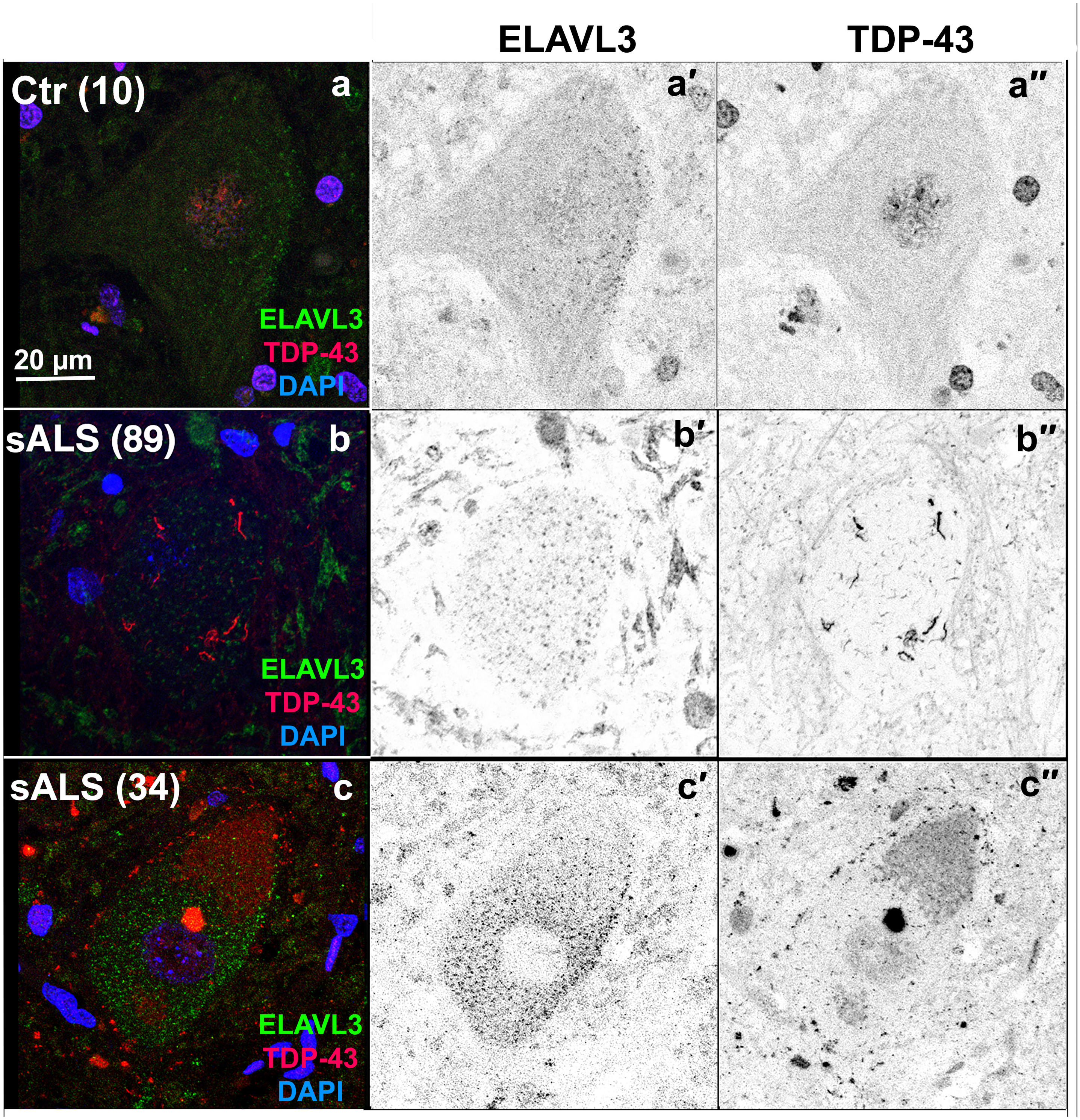

Supplementary Figure 9. ELAVL3 inclusions do not co-localized with TDP-43 aggregates

Supplementary Figure 9. ELAVL3 inclusions do not co-localized with TDP-43 aggregates. A. Control motor neuron has nuclear ELAVL3 and nuclear TDP-43. B-C. Cytoplasmic inclusions of ELAVL3 and TDP-43 in motor neurons do not co-localize. [Scale bar 10μm.]

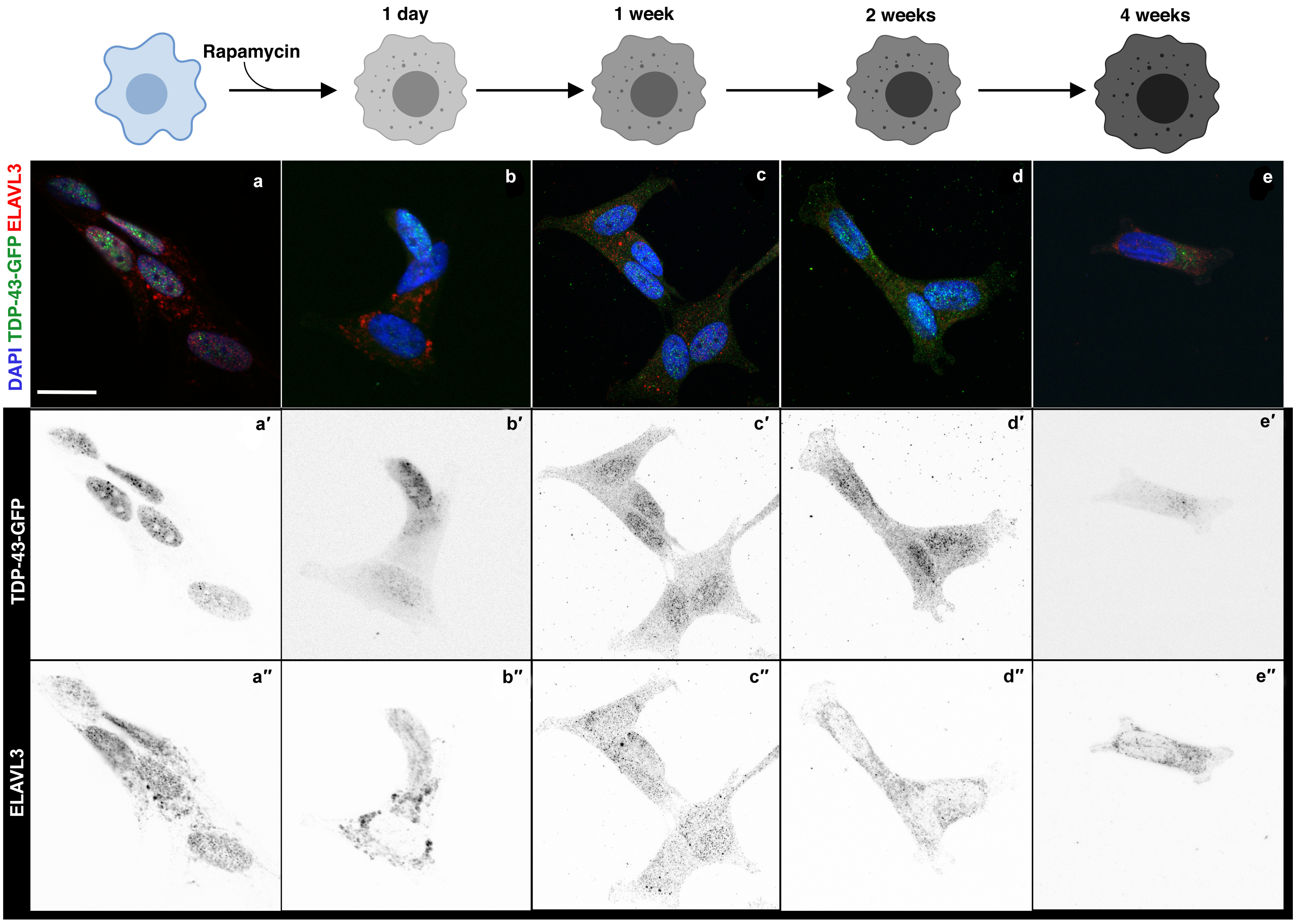

Supplementary Figure 10. Nuclear clearance of ELAVL3 is prior to TDP-43 mis-localization in cellular model.

Supplementary Figure 10. Nuclear clearance of ELAVL3 is prior to TDP-43 mis-localization in cellular model. Cell-cycle arressted SH-SY5Y cells are stressed by rapamycin and TDP-43 and ELAVL3 are studied over time at unstressed (A), 1 day (B), 1 week (C), 2 weeks (D) and 4 weeks (E) after rapamycin treatment. ELAVL3 abnormalities appear early and TDP-43 late.

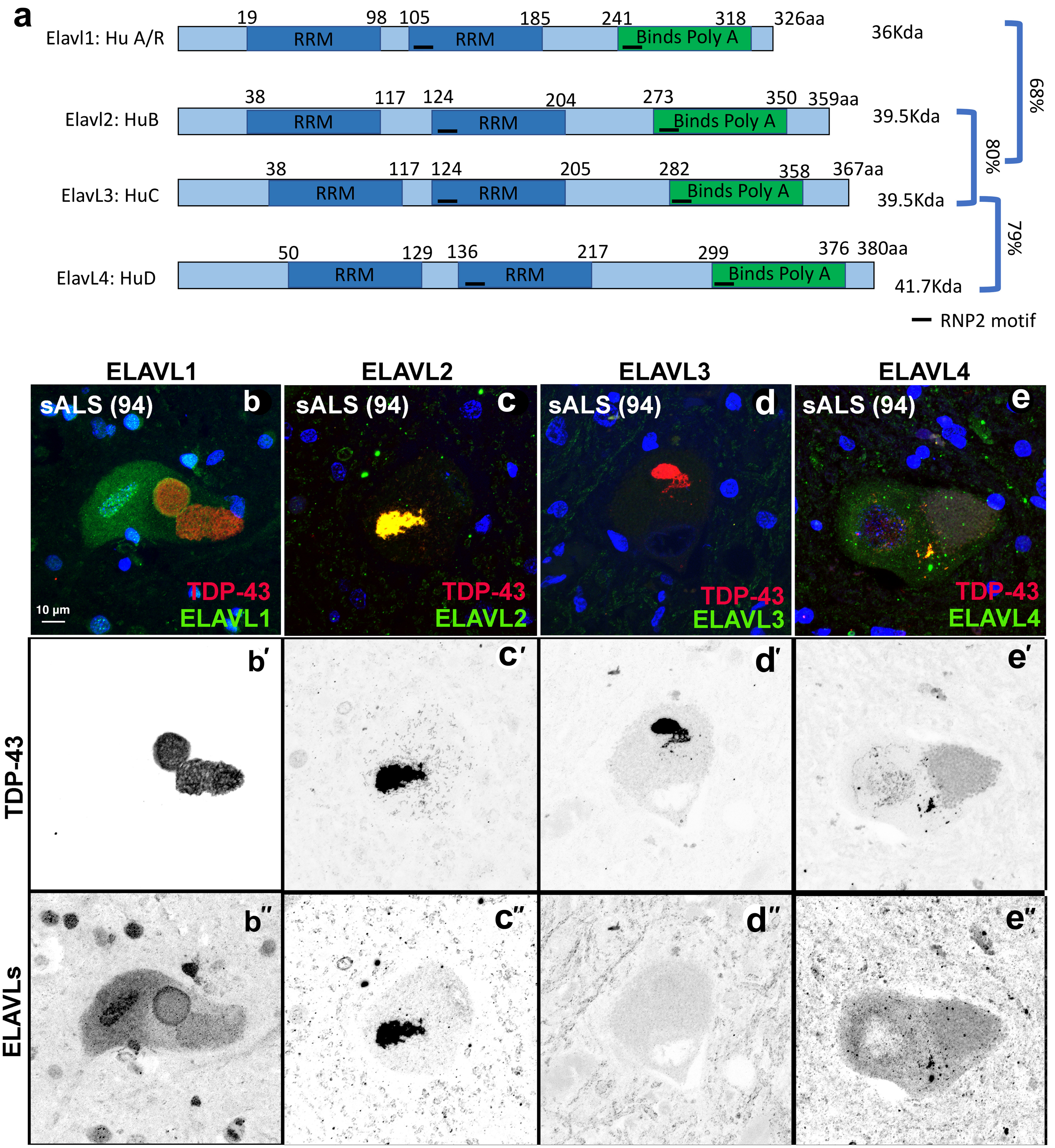

Supplementary Figure 11. ELAVLs protein behave different within the same nervous system.

Supplementary Figure 11. ELAVLs protein behave different within the same nervous system. A. Scheme of DNA sequence homology between different ELAVL3 family members. B. Staining of ELAVL1, 2, 3 and 4 in the same patient at contiguous sections. ELAVL1 is seen encapsulating TDP-43 aggregate. ELAVL2 co-localized with TDP-43 aggregates. ELAVL3 is nuclear depleted. ELAVL4 is depleted in the nucleus and aggregated in the cytoplasm where it partially co-localizes with TDP-43.

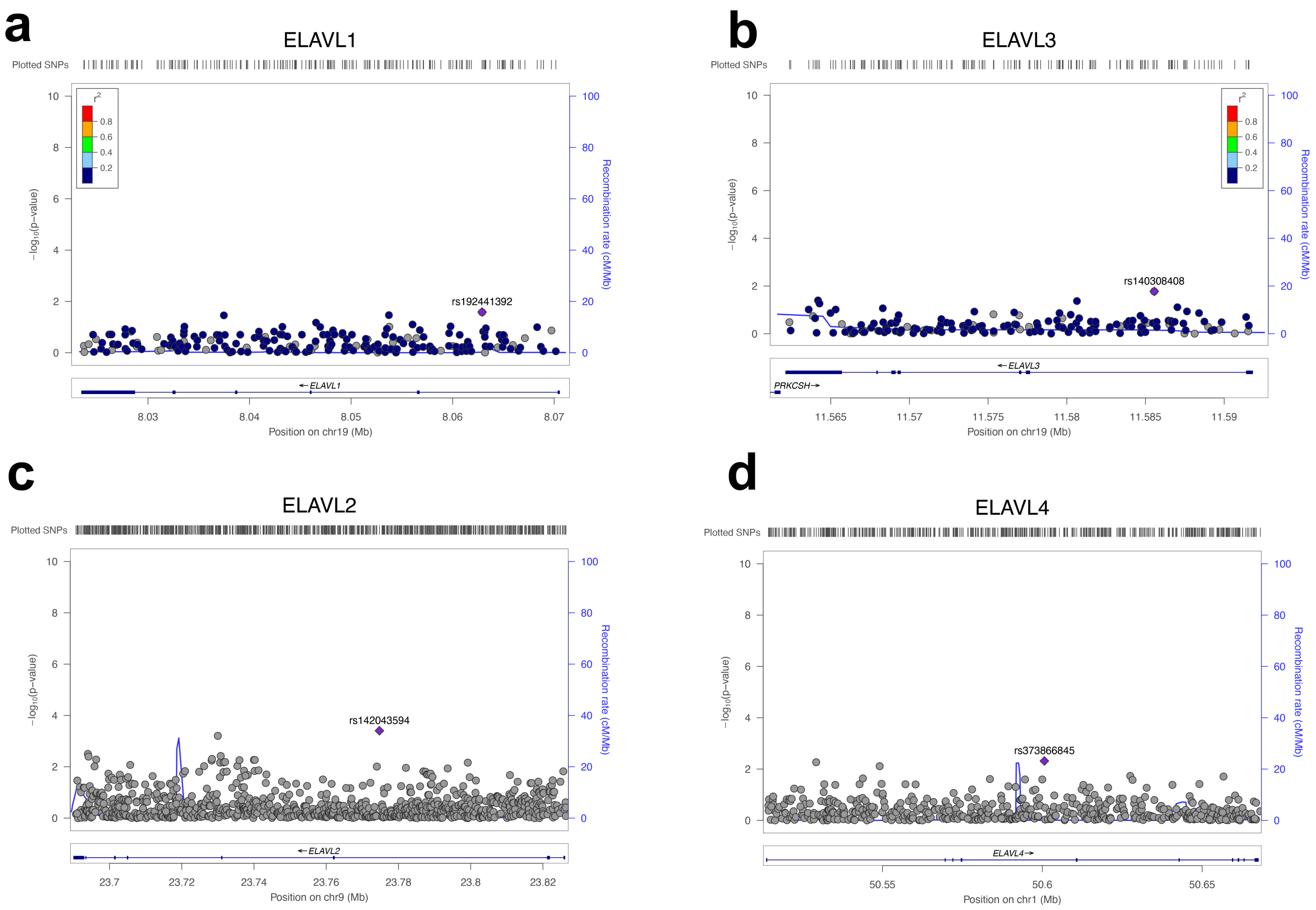

Supplementary Figure 12. Genetic variants of ELAVL family members were not found to be associated with sporadic ALS.

Supplementary Figure 12. Genetic variants of ELAVL family members were not found to be associated with sporadic ALS. Regional association plots show association statistics of variants with minor allele frequency > 0.01 for A) ELAVL1, B) ELAVL2, C) ELAVL3 and D) ELAVL4. Recombination rates and LD information across each gene was overlaid based on the population structure of 1000 Genomes (hg19). Each dot is a variant colored by LD R^2 with respect to the index variant with the smallest p-value within the gene (purple dot). The y-axis represents the $-\log_{10}$(p-value) and the x-axis gives the genomic coordinates in hg19. Threshold for significance was set at 5×10^{-8}. None of the common variants tested pass the significance threshold of $p = 5 \times 10^{-8}$, implying that these variants do not contribute significantly to risk in the sporadic ALS cohort.

Suppl. Table 1. Patients stained with ELAVLs

ID	DIAGNOSIS		Atxn2	CLINICAL INFORMATION			
Patient No.	Primary Diagnosis	Secondary Diagnosis	Intemediate length repeat expansion	Age	Gender	Site of Onset	Disease Course (yrs)
9	ALS			81	Male	Bulbar	2
15	FALS	A4V		65	Female	Arm	1.2
18	SALS			80	Female	Bulbar	2
19	Control	Parkinson's disease		80	Female	NA	NA
20	Control	Basilar CVA		38	Male	NA	NA
21	SALS			84	Male	Respiratory & hand	1.2
22	SALS			72	Male	Respiratory & hand	3.25
32	SALS		Yes	71	Male	Respiratory & trunk	1.5
34	SALS			81	Female	Bulbar	1
36	SALS			73	Male	Leg	1
37	Control	Vfib Arrest		57	Male	NA	NA
39	Control	Aortic Dissection & Multiple system failure	Yes	77	Male	NA	NA
44	Control	Liver Failure		80	Female	NA	NA
60	SALS			58	Female	Bulbar	3
62	SALS			52	Male	Arm	1.67
65	Control		Yes	82	Male	NA	NA
83	Control	Sepsis, myeloma, ARF, DM		63	Female	NA	NA
88	Control	CVA, multisystem failure		78	Female	NA	NA
89	SALS			36	Male	Bulbar	3
90	FALS/FTD	C9orf72 Expansion		52	Male	Bulbar/FTD	4
91	FALS	C9orf72 Expansion		61	Male	Bulbar	3.5
92	SALS		Yes	61	Female	Leg	1.75
94	SALS			62	Male	Bulbar	7
96	SALS/FTD			67	Female	Bulbar	2.5
98	FALS	C9orf72 Expansion		69	Female	Bulbar	1.5
99	SALS			70	Female	Bulbar	2.5
101	FALS	SOD1 (A4V)		68	Male	Leg	1.25
103	Control	Respiratory Failure		92	Female	N/A	N/A

**107**	SALS			74	Male	Trunk	0.75
**108**	SALS		Yes	69	Male	Leg	9
**114**	FALS (SOD1)	A4V		48	Male	Leg	1.2
**115**	Control			94	Male	NA	NA
**117**	SALS	C9orf72 Expansion		66	Female	bulbar	1
**118**	SALS			56	Female	bulbar	2
**120**	SALS			64	Male	bulbar	7.5
**124**	FALS	SOD1(c.317C>t;p. Ser106LEu) Also has ARHGEF28 C4307A>G; p.GLu1436Gly		45	Female	Left foot	2.25
**126**	FALS	C9orf72		70	Male	Right hand	1.75 ?
**128**	SALS			69	Female	Right Foot	2
129	FALS	SOD1 / c.14C>T P.Ala5vAL		69	Male	Left leg/ Left arm	9 month
**131**	Control			56	Male	NA	NA
133	Control			76	Male	NA	NA
132	SALS			64	Female	Right arm	10-12
135	SALS			77	Male	Respiratory/Thoracic	6.5

Id. of patients stained for each ELAVL

HuR	HuB	HuC	HuD
9	34	15	9
18	62	**19**	18
**19**	**83**	**21**	**19**
20	92	22	20
**21**	94	32	21
**83**	101	34	22
**94**	114	37	32
**101**	117	39	34
107	120	60	36
	124	62	37
	126	63	39
	65	65	44
	39	**83**	60
		88	62
		89	65
		91	83
		92	88
		94	89
		96	90
		98	91
		**101**	92
		108	94
		114	96
		118	98
		120	99
		124	101
		126	103
			107
			108
			114
			117
			118
			120
			124
			126

HuR & HuD
**HuC&HuR**
HuC & HuD
HuC & HuB
All

Western blot

#line	Sample	Code	ul
1	Marker		3
2	Control	131	30
3	Control	133	30
4	ALS cervical	128	30
5	sALS	118	30
6	sALS	132	30
7	sALS	135	30
8	C9	120	30
9	C9	117	30
10	SOD1	129	30
11	SOD1	114	30
12	SOD1/thoracic	124	30

Suppl. Table 2. ELAVLs antibodies and antigens:

HuA/R
https://www.ptglab.com/products/HuR-Antibody-11910-1-AP.htm

Peptide Sequence
MSNGYEDHMAEDCRGDIGRTNLIVNYLPQNMTQDELRSLFSSIGEVESAKLIRDKVAGHSLGYGFVNYVTAKDAERAINTLNGLRL
QSKTIKVSYARPSSEVIKDANLYISGLPRTMTQKDVEDMFSRFGRIINSRVLVDQTTGLSRGVAFIRFDKRSEAEEAITSFNGHKPPGSS
EPITVKFAANPNQNKNVALLSQLYHSPARRFGGPVHHQAQRFRFSPMGVDHMSGLSGVNVPGNASSGWCIFIYNLGQDADEGIL
WQMFGPFGAVTNVKVIRDFNTNKCKGFGFVTMTNYEEAAMAIASLNGYRLGDKILQVSFKTNKSHK

(1-326 aa encoded by BC003376)

HuB
https://www.ptglab.com/products/ELAVL2-Antibody-67097-1-Ig.htm

Peptide Sequence
LSNGPTCNNTANGPTTINNNCSSPVDSGNTEDSKTNLIVNYLPQNMTQEELKSLFGSIGEIESCKLVRDKITGQSLGYGFVNYIDPKD
AEKAINTLNGLRLQTKTIKVSYARPSSASIRDANLYVSGLPKTMTQKELEQLFSQYGRIITSRILVDQVTGISRGVGFIRFDKRIEAEEAIK
GLNGQKPPGATEPITVKFANNPSQKTNQAILSQLYQSPNRRYPGPLAQQAQRFRFSPMTIDGMTSLAGINIPGHPGTGWCIFVYNL
APDADESILWQMFGPFGAVTNVKVIRDFNTNKCKGFGFVTMTNYDEAAMAIASLNGYRLGDRVLQVSFKTNKTHKA

(5-346 aa encoded by BC030692

HuC
https://www.ptglab.com/products/ELAVL3-Antibody-55047-1-AP.htm

Peptide Sequence PALPNGPLLGTNGATDD

(19-36aa)

HuD
https://www.ptglab.com/products/ELAVL4-Fusion-Protein-ag21830.htm

Peptide Sequence MVMIISTMEPQVSNGPTSNTSNGPSSNNRNCPSPMQTGAT

(1-40 aa encoded by BC036071)

HuC LsBio
https://www.lsbio.com/antibodies/elavl3-antibody-huc-antibody-ihc-ip-wb-western-ls-c408905/421270

Peptide Sequence

MVTQILGAMESQVGGGPAGPALPNGPLLGTNGATDDSKTNLIVNYLPQNMTQDEFKSLFGSIGDIESCKLVRDKITGQSLGYGFVN
YSDPNDADKAINTL

A synthetic peptide corresponding to a sequence within amino acids 1-100 of human ELAVL3 (NP_001411.2).

Suppl. Table 3. Missregulated RBPs in Rabin et al 2009 and Krach el at 2018

	**Rabin**			**Krach**		
	**Log2Fold-Change**	**FC**	p-value	**Log2Fold-Change**	**FC**	**p-value**
TARDBP	1.13888	2.20210002	0.205076	1.15274707	2.22336849	0.22677708
FUS	-1.01705	0.4941257	0.812063	1.75572026	3.3769487	0.98454659
SOD1	1.03439	2.04824742	0.74499	1.67596265	3.19532495	0.16581409
SOD3	1.02695	2.03771177	0.623243	1.54033377	2.90861788	1.73E-06
TAF15	1.03747	2.05262488	0.638278	1.55647025	2.94133325	0.2036429
HNRNPA1	1.21893	2.32774012	0.0200043	1.0139625	2.01945011	0.01624574
HNRNPA2B1	1.0292	2.04089223	0.725174	1.6531	3.14508716	0.04932847
EWSR1	1.14487	2.21126204	0.258623	1.1963363	2.29156991	0.30126803
MATR3	-1.00101	0.49965008	0.990768	1.98724259	3.96478488	0.15437662
TBK1	1.1679	2.24684406	0.259341	1.19693184	2.29251606	0.16255541
ANXA11	1.0937	2.13420683	0.118415	1.08554159	2.122172	0.41506731
ELAVL3	-1.2581	0.41809422	0.00090006	-1.1831576	0.44038658	0.00000004
ELAVL1	1.0069	2.00958834	0.933523	-0.3082003	0.80764864	0.06679774
ELAVL2	-1.4208	0.37350514	0.0818584	-0.2537431	0.83871752	0.17983045
ELAVL4	-1.22967	0.42641497	0.337809	-0.1393821	0.90790793	0.50750021
NOL8	0.20465408	1.15241	0.0554378	0.59183859	1.50716628	0.00049214

Suppl. Table 4. Quantification of neurons with

ELAVL3

Patients	total neurons		ELALV3				
		nuclear			cytoplasmic		
		nuclear signal	nothing visible	dots	speckles	fiblils	rounds
94	10	4	10				
96	9	2	7	2			
21	6	3	6				
63	7	2	7				
22	16	10	16				
62	6	1	5	1			
36	21	11	13	8			
34	35	4	28	6			1
43	8	3	5	3			
32	20	3	9	11			
92	23	4	7	16			
126 (C9 orf72) not included	17	6	9	8			
98 (C9 orf72) not included	8	4	8				
SUM	186	57	130	55			1

TDP43

Patients	total neurons		TDP-43					
		nuclear			cytoplasmic			
		nuclear signal	nothing visible including with nuclear signal	dots	speckles	fiblils	rounds	
94	10	7	9				1	round hive like
96	9	4	5		4			TDP-43 speckles & fibrils within same neurons
21	6	3	6					
63	7	3	7					
22	16	16	11		5			
62	6	2	2	2		2		
36	21	18	20			1		
34	35	22	25		7	2	1	round filamentous like
43	8	4	4		4			TDP-43 speckles & fibrils within same neurons
32	20	16	5		15			
92	23	4	8		11	2	2	
126 (C9 orf72) not included	17	7	7		4	6		
98 (C9 orf72) not included	8	5	7			1		
SUM	186	111	116	2	50	14	4	

Summary ELAVL3 and TDP-43

Patients	% nuclear loss of ELAVL3	% cytomplasmic inclusions	% nuclear loss of tdp-43	% cytomplasmic inclusions
94	60.00	0.00	30.00	10.00
96	77.78	22.22	55.56	44.44
21	50.00	0.00	50.00	0.00
63	71.43	0.00	57.14	0.00
22	37.50	0.00	0.00	31.25
62	83.33	16.67	66.67	66.67
36	47.62	38.10	14.29	4.76
34	88.57	20.00	37.14	28.57
43	62.50	37.50	50.00	50.00
32	85.00	55.00	20.00	75.00
92	82.61	69.57	82.61	65.22
126	64.71	47.06	58.82	58.82
98	50.00	0.00	37.50	12.50
	66.23	23.55	43.06	34.40

	ELAVL3 LOF	ELAVL3 GOF	*Marginal Row Totals*
**Neurons with problems**	114 (81) [13.44]	48 (81) [13.44]	162
**Neurons without problems**	47 (80) [13.61]	113 (80) [13.61]	160
Marginal Column Totals	161	161	322 (Grand Total)

The chi-square statistic is 54.1139. The *p*-value is < 0.00001. Significant at $p < .05$.

The chi-square statistic with Yates correction is 52.4865. The *p*-value is < 0.00001. Significant at $p < .05$.

	TDP-43 LOF	TDP-43 GOF	*Marginal Row Totals*
**Neurons with problems**	74 (66.5) [0.85]	59 (66.5) [0.85]	133
**Neurons without problems**	87 (94.5) [0.6]	102 (94.5) [0.6]	189
Marginal Column Totals	161	161	322 (Grand Total)

The chi-square statistic is 2.8822. The *p*-value is .089563. *Not* significant at $p < .05$.

The chi-square statistic with Yates correction is 2.5107. The *p*-value is .113074. *Not* significant at $p < .05$.

Suppl. Table 5. Common down-regulated RNAs

https://bioinfogp.cnb.csic.es/tools/venny/	elife-10421-supp1-v2 targets brain	1D nELAVL targets brain	Scheckel et al elife

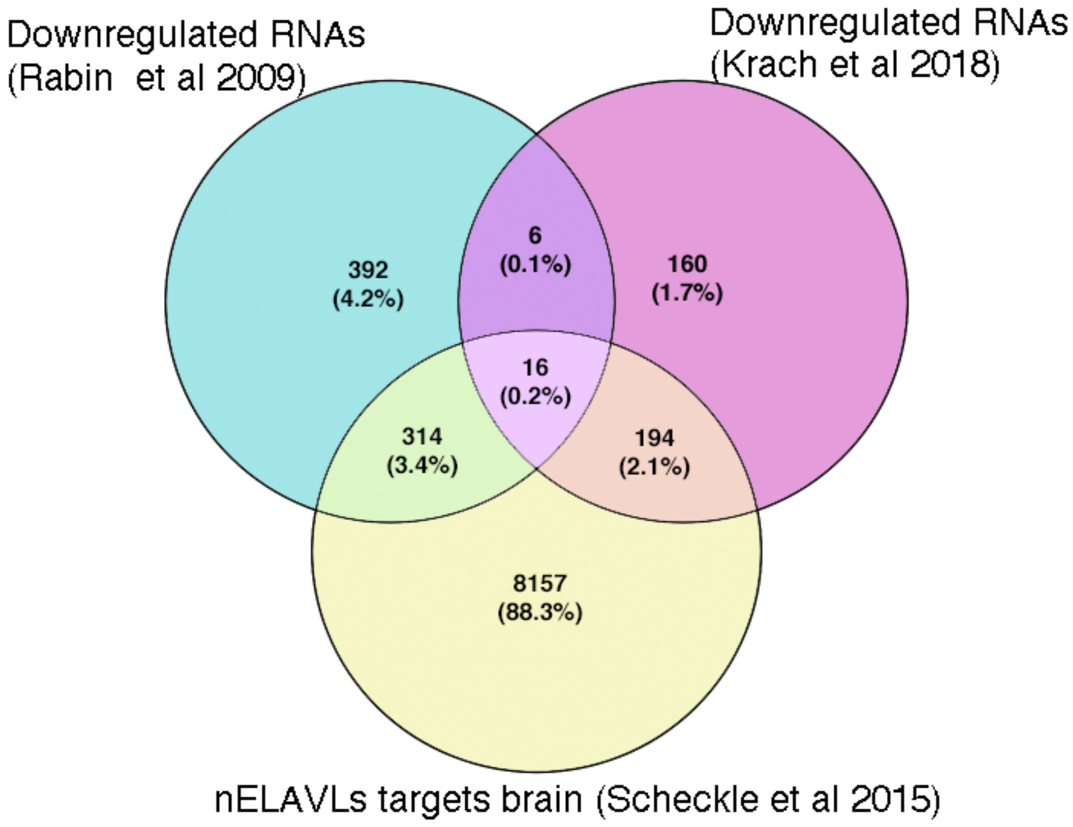

6 common elements in "Rabin downreg" and "Krach downreg": RXRG RAB3D CORO2A NNAT
KCNK9 WDR63

314 common elements in "Rabin downreg" and "nELAVL clip": HSN2 HMGCS1 KIF5A
PENK RGS4 SLC45A4 SNCG CPLX2 DHCR24 BCAT1 SNCB KRT222P
SYP EIF5A2 MGC48628 NRSN1 TMEM97 NMNAT2 TAC1 C3orf57 ZNF721
MCF2 SCN2A RLBP1L1 SLC12A5 FREQ TMEM130 FAM135B RIMS2
TUBA8 SETD8 AP3B2 RNF111 BICD1 GABRA5 DYNC1I1 GRIN2B SNRPA1
ADAM19 PPP1R2 PRKCZ DLG3 ZMAT4 TRPC5 PCSK2 TRIM52 VTI1A GAD2 RHBDL3
JPH3 FXYD7 NELL1 IGF1 MXRA7 EHD3 SLC25A12 DCC C6orf138 MAST1
SUSD1 SLITRK3 AP1S1 ATRNL1 MCTP1 CCDC64 MCF2L2 SSTR1 PRKG2 NRG1 CRYM
AFF2 NPAL2 DLG4 NCAN C15orf17 C6orf168 RPRM FAM57B
KIAA0513 ADCYAP1 RAB15 P2RX5 L1CAM C1orf163 GRIN2A ADD2 TRO
PLD5 PPAP2C CSPG5 GRIA1 KCNB2 CNTN4 GREM2 PACSIN1 CPLX1 NRGN
C12orf53 CBLN2 TMEM169 ANK1 GRAMD1B SNPH ANKRD52 RBP4
STMN3 EPB49 PRMT8 CNNM1 TMEM59L APBA1 ABCC5 TMEM177 ARPP-21
FLJ43276 FSTL4 ATP10B PAQR9 SYN3 KCNA4 NDRG4 GPI GRM5 C9orf68 ATP2B3

HS3ST2 MPV17L CADM3 TAC3 KLC2 RASGRF1 ACSL6 DTNB CACNA1E
STYK1 FBXW4 GPLD1 NIPSNAP1 OGDHL SYN1 FAM40B C21orf56
SLC24A3 DOK6 GRIN3A RNMTL1 ADCK1 CLTB CPEB1 SPSB1 PPP2R2B
SH3GLB2 CYCS DSCAM WDR4 HAPLN4 KLHL7 SYNJ2 ACTN2 KCNMB2
TF RDHE2 HSPA14 FAM80A ZNF282 TMEM175 BRSK1 HMOX2 OSBP2 GRIK1
ARHGAP28 CLSTN3 ANKRD16 MAGI1 DACH1 KIAA1409 CHGA SEZ6L TRHDE
CACNB1 PCDH21 RIMS1 SNX12 NEURL WDR85 TTC5 OLFM1 NPTX1 CDK5R1
SYT7 CD99L2 RAB11FIP3 CIT PTPN5 TGFA SPTB KCNAB2 GRM1 BDH1
CREB5 HS3ST4 ATXN7L1 CACNG4 PGBD5 ARMCX2 GTPBP3 KATNB1
VDAC2 DACH2 PGBD1 RAPGEFL1 CHD5 HYDIN SEMA7A STK35 OLFM2 KCNS3
TOX2 ATP2B2 CAMKV KCNH7 MFSD4 TMED8 SMUG1 SMYD5 CAMK2A SHANK2
TNRC4 ZNF786 GRM7 AFF3 USP5 HIP1R HS6ST2 RNF207 ZNF266 CALM3 ICA1
EPB41L1 KIAA0802 HMGB3 CACNA1B KCNC3 PNKD NPTX2 KIAA0427
SLC35F3 PDXP SORCS3 CYP2E1 USP12 VSTM2L BRSK2 TMEM39B GGA3
ACACA POLRMT DNHD1 CRMP1 TPM3 CXorf34 ZMYND8 SMARCD1 PIN1
SRGAP3 AAMP DPYSL4 PRKCE GREB1 SLIT3 FOXP2 ZC3H3 APBB1 C9orf86 EPHB2 NUPL2
PPFIA3 SLC35A1 LMTK2 SETD6 CDYL2 SPPL2B GABRA4 BCL11A MVD
ZDHHC24 CENTG3 C20orf112 ARHGEF11 PLXNA1 HSD11B1L CSMD1
NLGN3 SEC14L5 EVI5L THOC5 OSBPL10 ATP13A2 ADCY5 LIG3 KCNQ3
PCNT RALGPS1 DUSP26 CCRK ZER1 CCM2 CACNA1A CACNA1D
DSCAML1 ADCY1 LSM4 DAZAP1 IGSF11

16 common elements in "Rabin downreg" "Krach downreg" and "nELAVL clip": STMN2
PRUNE2 ATP8A2 MAP2 ADARB1 ENTPD3 VSTM2A MAP3K9
HSPA12A PCYT1B UBE2O ELAVL3 HECW1 SEMA3A UBE3C SLC8A3

194 common elements in "Krach downreg" and "nELAVL clip": ANO3 CUX2 RNFT2 MADD
CLCN5 DOPEY2 SLITRK1 KIAA1841 SLC7A8 SACS CRB1 TNS1 ISOC1 USP13
MAP2K4 XYLB UCK2 KCNA1 DENND1B PI4K2A MMP16 TAPT1 SNURF SIL1
PCP4L1 CAMK1D IFT122 MMAA RFPL1S STX1B EPB41 UTP15 SLC41A2
MAPK8IP3 VPS13A HTT PRKRIP1 RYR2 IPO11 PDK3 DGKI HECW2 CHRM2
RNF157 POLR1A MAST3 LRP12 ZKSCAN2 NOS1AP SLC16A7 RCC2
MYCBP2 LRP8 PSD3 ANKRD50 CLCN4 GNB5 GRIP1 DLGAP1 SARS ENTPD4
PPM1H CPEB3 MAPK10 LNPEP PFKP HERC1 MYO9A TLN2 DMXL2 PSME4 HKR1
SLIT2 HEATR5B OSBPL9 PEG10 INADL REPS2 NSFL1C SLC9A6 TBC1D9 ZNF273
FUNDC2 RAB11FIP4 CLASP2 SYN2 TUBA1B TTBK2 HK1 CLASP1 DRG1
GRIA2 NALCN GSPT2 KIAA1377 PDPK1 DENND5B ANK2 PITPNA EXOC6B WWP1
TM7SF3 KIAA1715 POLR3A YBX1 STK38 ZNF184 PFKFB2 RNF141 CETN3 SLC8A1
SORBS1 ANKRD40 SOCS6 PHLDA1 CNOT6L CTSC RPS13 RPS25 RPL26 CCDC50 MYH9
KLF3 PHC3 HMGN3 RPL13A SETD7 TMEM2 C11orf30 TWSG1 RPL38 MIER3 SNHG6
KIAA1598 GPT2 VEZF1 SRF STMN1 RAB31 MITD1 SCN7A RPL34 PTPRF
ARHGAP26 TRIM8 HNRNPF PTPRA ZMIZ1 CLK4 BBX DMD EPC1 GAS5
INTS3 RPL27 RNF38 CREBZF FOXN3 PIP4K2A RND3 PABPC1 RPL27A MCL1 PTP4A2
QSER1 TRMT11 DECR1 NPC2 HP1BP3 TPT1 IQGAP1 SEC11A CTNNB1
RPS6KA2 ASAH1 RPL4 RPL9 RPS20 TCEA1 MED29 TIMP2 RPL22 RPS24 S100B
ARHGAP12 CNTLN AK2 ADAM10 RPL5 ZNF226 RPL15 ARHGEF6 ANXA5
PEA15

Suppl. Table 6. Common down missregulated RNAs

elife-10421-supp1-v2 targets brain	1D nELAVL targets brain	Scheckel et al elife

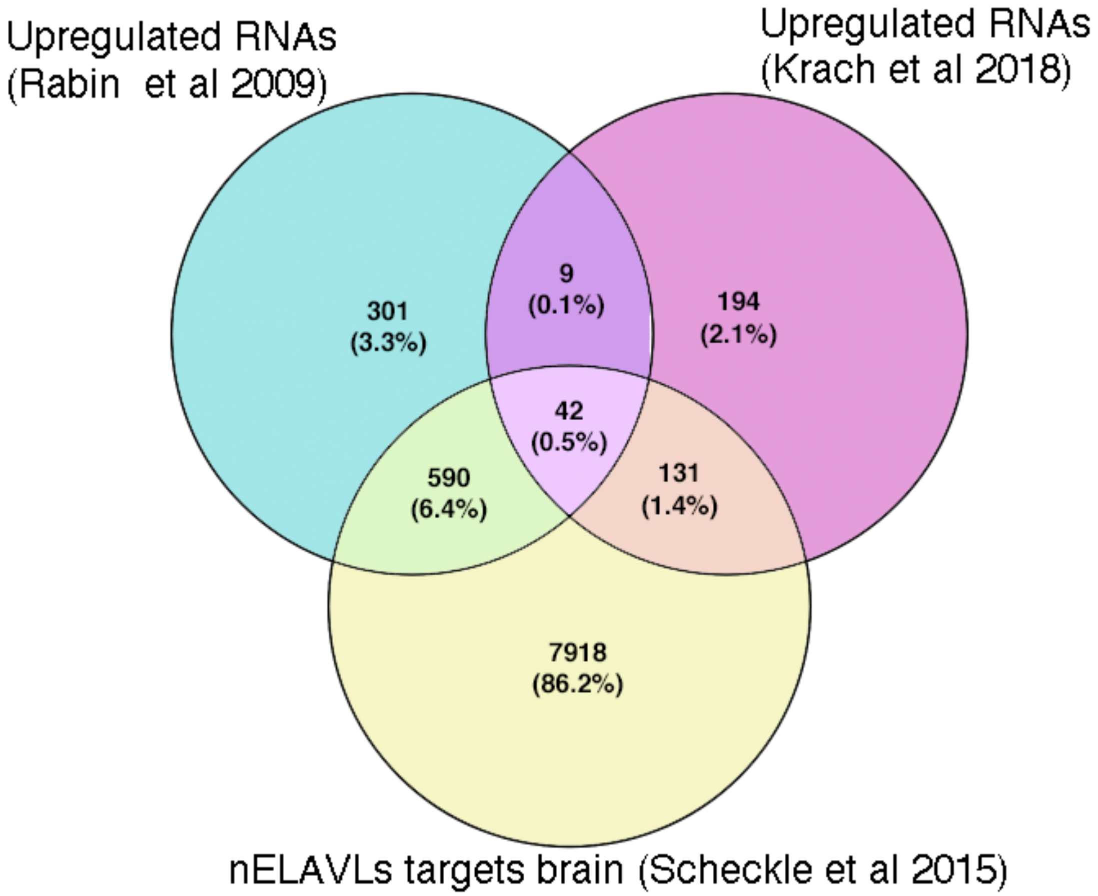

9 common elements in "Rabin upreg" and "Krach upreg": GPNMB HERPUD1 CYP4F11 C1S CHI3L1 PRKD3 RCN1 VAT1 NOL8

590 common elements in "Rabin upreg" and "nELAVL clip": ADCY8 TSHZ2 AQP1 HADH FNDC3B SSPN OGFRL1 LGALS3 SLC7A2 ERCC4 IGF2R CD44 SP1 FGF2 CYP1B1 CALCRL CXCL16 SMNDC1 ANTXR2 KATNAL1 EDNRB RIC8A MAPKAPK5 GRAMD3 KIAA0174 CPNE8 PGGT1B CD81 SERP1 CLIC4 ALDH1A1 PLSCR4 ASCC3L1 MFAP3L C9orf5 PPM1D MAN1C1 DLG5 PLEKHA5 CNOT1 SFT2D1 TJP2 SOCS4 TRPS1 GFAP AQP4 NUP35 LHFP EIF4EBP2 TMEM65 C9orf103 WDR51B DNAJC1 LAMA4 DDAH1 SNX7 NTRK3 STK4 ATF7 HIST1H2AC PIK3C2A SCMH1 NOTCH2 MSN SLC16A9 MFSD1 CHUK MAP3K5 PXMP3 ITGA6 SMARCA1 ATP6V0E1 C12orf35 CXorf39 MAN1A1 COLEC12 UTRN SLC1A3 CAT DLST NUMB SMAD4 ELP4 ACSBG1

LASP1	TRAF6	WWTR1		NCOR1	UST	COG5	FUT10	ARSB	MED13L		VIM
SPG20	ZMYM1	PCDHB4		SBF2	C9orf61	KLHL23	ZFX	SDCBP	ANKFY1	CTNND2	
TEX2	HSPB8	DNAJC3	PROS1	CRLS1	BNC2	TOB1	CUGBP2		PRDX6	NCOA1	DAAM1
DUSP1	C5orf33	ABLIM1	LMAN1	GPRC5B		PPFIA1	PAQR3	ANTXR1		TOR1AIP1	
USP53	STK38L	TBC1D1	ZEB1	CYBRD1	GPR177	SPG11	SEC23B	ARFGAP3		LMO4	GNA13
THOC2	SF3B3	C10orf46		RFX3	PLOD2	RPL30	SMEK2	NR4A2	MPZL1	LGR4	SRGAP1
OSBPL11		NMT1	ABI1	ZFP36L1		PADI2	TPST1	SFRS4	FNBP1L	FARP1	
PRPF38A		PARD3	SLC39A12		GNAI3	C6orf145		C14orf147		PSAP	DPYSL3
DCN	RGMA	PMP2	PTPRZ1	LAMA2	APAF1	KCTD20	RBM4	RBM12	TMEM168		PPP2CB
CNTN1	GPM6A	METTL9	STOM	TICAM2	PRKDC	PHF20	FBXL7	DYNLT1	ELF1	UXS1	VCL
IVD	CRYL1	ITPR2	UBE2E1	GOLGA8A		ALDH9A1		ROCK1	ZNF436	MTM1	GTF2E1
GIT2	STAG2	C1orf77	ZC3HAV1		DIAPH2	SLC3A2	MED17	HIF1A	MANEA	PRPF4B	CNIH4
MTMR3	TXNDC11		CCDC45	PARP14	SMC5	RUFY1	PRPF40A		GFPT2	MTA2	STAT3
INADL	CTSB	GRINL1A		RIPK2	TMEM123		HPS3	VRK2	NEBL	C9orf64	USP6NL
ELMO2	PAWR	SCYL2	SAMHD1		FAM63B		HS2ST1	TMOD3	C6orf192		AMOT
ST6GALNAC3		LCORL	GABRG1		DBC1	SMARCB1		WDR45L		STAG1	FADS2
RAB8A	FMN2	PPP4R1	TXNDC4		CLIP1	COPB1	ABR	FZD4	ZFAND3		DHRS3
PDPR	KIAA1429		ZRANB1		NIN	BFAR	DARS	HNRNPA1		CHPT1	GNS
PPP1R1A		APPBP2	CCDC59	ARFIP1	LRIG1	PDGFC	LSM14A		ST5	CORO1C	
TXNDC1		SFRS2IP	TMEM136		ASCC1	C10orf104		SESN3	CTCF	DAD1	
KIAA1199		FAT	TGFBR2	TEAD1	BMPR1B		PDIA6	SAFB	UTX	RAB5C	CNN3
ELF2	DTNA	USP3	USP47	MAP3K7IP2		ATF6	RASSF8	TPP1	C13orf23		GLIPR1
STAU1	REV1	C11orf46		ITM2B	ATP1B2	CCPG1	NUP107		SS18	SNF1LK	OPHN1
SFRS7	GOLM1	TBL1X	GNB4	MANBA	SLC31A1		ARNT	CASP3	ZNF614	GNB2L1	USP28
MYST3	KRCC1	SLC33A1		CCDC47	LRP1	YME1L1	CRTAP	FBLN5	CASP8AP2		CLU
CYB5A	ATP2B4	PDPN	BMPR1A		RBBP8	NXT2	CLNS1A	H3F3B	HIST1H2AE		PHIP
GLT8D3	BRWD3	UBE2R2	NEO1	TOX4	ADAM17		WHSC1L1		SERPINE2		DDB2
CFDP1	MAPKAP1		HSP90B1		PCTK2	PPP2R5C		ASPH	YWHAZ	ZNF706	REEP3
VCAN	SLC16A2		COMT	C10orf119		SMOC1	DUSP16	CENTB2	FAM49A		EIF4E2
RP2	ARHGEF3		ZHX1	GABARAP		YAP1	SMC1A	TGFB2	OSGIN2	DENND4C	
C17orf71		ZCCHC2	AZIN1	BBS2	CPNE3	SORL1	TMTC3	GBAS	RER1	JAK2	RDX
CD9	TMEM9B		ADIPOR1		GLG1	CCDC117		CNOT6	PPP2R5A		TMED5
SLMO2	C16orf61		BET1	ACTN1	CFLAR	FAM92A1		DKC1	ZNF227	NUCB2	
MINPP1		TMED10		GPC6	DPYD	FAM118B		TTC19	PNPLA8	CRNKL1	
MOBKL1A		NFE2L2	LIX1	SOD2	ACVR2A		LAMB1	RDH10	RBL2	BAALC	FYN
CHIC2	LTA4H	SMAD9	CLK1	C14orf135		EIF3EIP	APPL2	ADCY2	LGMN	SERBP1	
CGGBP1		CAV1	ACOX1	CALU	FBXL5	ZNF616	CTSA	HSDL2	SLC23A2		RPN2
TNKS2	CCT3	C14orf166		STK3	HIPK1	RANBP3L		POLA1	ADRB1	VCP	AKAP8L
MAPK4	DHX9	CALR	SMU1	DKK3	TRIM68	JOSD1	DSC2	RAB28	TLE4	TMEM47	
TEGT	CPE	ACPL2	ZZZ3	LSM6	ADD3	SLC22A5		NFXL1	DNER	MTPN	SETD5
C8orf4	ERO1L	UNC13B		TANK	PRKRA	SLC35F5		CYP2U1	MGST1	PCGF5	NEK1
C1QTNF3		CYP27A1		SLC20A2		PPP1CB	ASCC3	PVRL3	FHL1	BMI1	IRF2
IFT81	CHST6	EIF2C4	SFRS3	TBCD	PFKFB3	CHD2	FUT11	RNASEH2B		PPP1R3C	
RSF1	SLC39A11		TUG1	YES1	FUBP1	ANXA4	DDIT4	HEATR1	CUL4A	CAPN2	MEX3C
EP300	FAM76B		WASF2	SMAD1	USP39	TNPO1	GPM6B	CD74	SEC31A	RSBN1	CAPN5
CTNNAL1		CAPRIN1		ANGEL1	KLF9	KIAA1012		CDC14B	PPP1CC	SLC39A14	
ARHGEF12		ITGAV	ARHGAP10		RAP1B	THSD4	LOC26010		MYLK	AATF	AHSA2
C14orf118		PDE7A	SMCHD1		CDH20	F3	ALDH18A1		PCDHB13		CHL1

C7orf46 MRPL50 FAM104A RASA2 LIPA RBBP6 MAP3K2 SOAT1
SEC61A1 MAP2K1IP1 SRP54 PTPRG EGLN3 PLXDC2 C10orf32 JARID1A
NSL1 THRB SDC2 PRCP MRPL3 AP2B1 RAB23 KIT CALCOCO2 SEL1L P4HA1
PTK2 ARHGEF10 EIF3H TUBB2B TM9SF3 PAPD4 DCAKD TRAM1 ZNFX1
MOBKL2B MLLT10 CPSF2

42 common elements in "Rabin upreg", Krach upreg and "nELAVL clip": PHKA1 MAOB SORBS1 GLIS3
ASAH1 PRKCA NPC2 CTNNA1 IQGAP1 SSFA2 AGT TWSG1 IL13RA1 PALLD
KAL1 NQO1 AHCYL1 RAB31 MCL1 GM2A CYFIP1 PARP4 LAMC1 NFKBIA LUZP2 B2M
CCDC50 ADAMTS1 ARHGEF6 ITM2C ANXA5 RNF38 THBS2 MYH9 TNFAIP1
HP1BP3 CTNNB1 CLK4 ERBB2IP ADAM10 RPS25 TIMP2

131 common elements in "Krach upreg" and "nELAVL clip": KLHL18 BTG1 EGR1 MAL
TRIM25 CDKN1A LPCAT2 FRMD5 ETS1 ELK4 SLC6A6 NHLRC3 GCH1 CLDN11 HMGB2
MAGT1 RELA GLDN GRIA1 PHF10 SLC25A37 LIMA1 FAT4 SAT1 MEF2C HSPA2
TCF12 COL12A1 PER3 C1orf63 ITGA2 CSNK1E SLC2A1 RGS5 ZNF577 MAF PHF21A
LPP PROX1 KLHL5 NPAS3 VGF NEK9 SENP1 SVIL CREB3L2 LIMK2 SNX5
PGRMC1 RPS3A CARD8 ZNF621 RPS4X CCNG2 FLNB PCDH1 RALGDS TIMP3 ZRSR2
YBX1 STK38 ZNF184 PFKFB2 RNF141 CETN3 SLC8A1 ANKRD40 SOCS6 PHLDA1 CNOT6L
CTSC RPS13 RPL26 KLF3 PHC3 HMGN3 RPL13A SETD7 TMEM2 C11orf30 RPL38
MIER3 SNHG6 KIAA1598 GPT2 VEZF1 SRF STMN1 MITD1 SCN7A RPL34 PTPRF
ARHGAP26 TRIM8 HNRNPF PTPRA ZMIZ1 BBX DMD EPC1 GAS5 INTS3
RPL27 CREBZF FOXN3 PIP4K2A RND3 PABPC1 RPL27A PTP4A2 QSER1 TRMT11
DECR1 TPT1 SEC11A RPS6KA2 RPL4 RPL9 RPS20 TCEA1 MED29 RPL22 RPS24
S100B ARHGAP12 CNTLN AK2 RPL5 ZNF226 RPL15 PEA15

Suppl. Table 7. Common down-regulated RNAs

Tam et al 2019 Table S3A A) List of genic targets bound by TDP-43 eCLIP

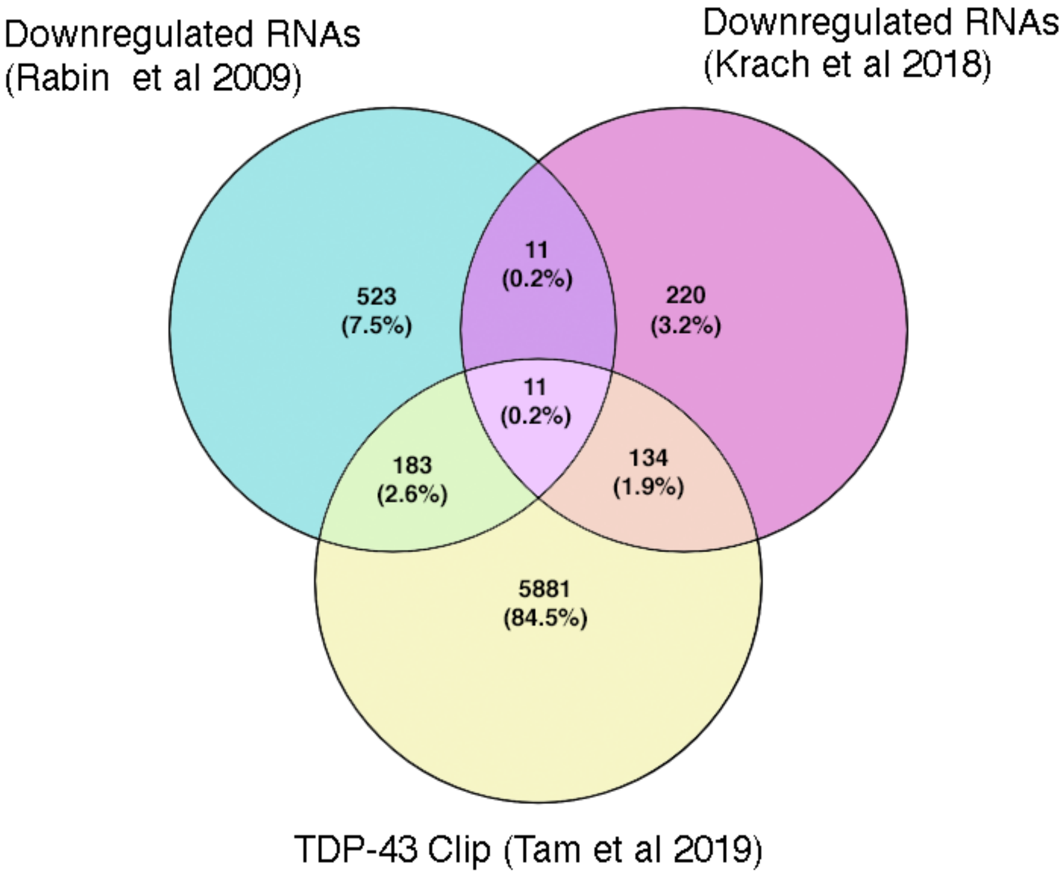

11 common elements in "Rabin downreg" and "Krach downreg": PRUNE2 RXRG RAB3D
ENTPD3 VSTM2A CORO2A PCYT1B NNAT HECW1 KCNK9 SEMA3A

183 common elements in "Rabin downreg" and "TDP-43 clip": CPLX2 DHCR24 BCAT1
TMEM97 HCN2 NMNAT2 ZNF721 MCF2 SCN2A PIN4 KLHL14 RIMS2 AP3B2
RNF111 BICD1 PCBP3 GRIN2B PRKCZ SLC18A1 TRPC5 VTI1A RHBDL3 SLC25A12
DCC MAST1 SUSD1 SLC29A4 ATRNL1 MCTP1 NRG1 CRYM DLG4 NCAN ISL1
P2RX5 L1CAM HIPK4 GRIN2A ADD2 TRO PAPPA PLD5 CNTN4 TMEM169
GRAMD1B SNPH ANKRD52 APBA1 ABCC5 FSTL4 NDRG4 GPI TRPC7 GRM5
NLRP8 HS3ST2 CADM3 FANCI RASGRF1 DTNB FBXW4 OGDHL SYN1 ACOT7 ZNF713
DOK6 PARD6G EMB PPP2R2B DKK2 GDAP1L1 CENPO WDR4 KLHL7
SYNJ2 ACTN2 KCNMB2 TMEM175 BRSK1 OSBP2 GRIK1 LIN28B CLSTN3 MAGI1
DACH1 DKK1 DNAH2 SEZ6L ZNF502 CACNB1 RIMS1 UNC119 SYT7 CD99L2
RAB11FIP3 SDC1 RMND1 SLC30A3 CIT ANKRD7 WDR18 IQCH CREB5
HS3ST4 ATXN7L1 CACNG4 PGBD5 GTPBP3 KATNB1 VDAC2 SPTBN2 PGBD1
RAPGEFL1 STK35 OLFM2 PCBP4 TOX2 PODXL2 SHANK2 ZNF786 GRM7 AFF3

HS6ST2 ZNF266 ICA1 EPB41L1 SHANK1 LIG1 CACNA1B STAC

SLC35F3 KEL GPR161 SORCS3 TACR3 USP12 VSTM2L BRSK2 ASB13

TMEM39B ACACA POLRMT DNHD1 AMACR CRMP1 ZMYND8 QRICH2 PIN1

SRGAP3 AAMP DPYSL4 PRKCE GREB1 SLIT3 GATA4 APBB1 EPHB2 NUPL2 PPFIA3

SLC35A1 LMTK2 SETD6 SPPL2B DNAH10 ARHGEF11 MARK4 CSMD1 THOC5

ATP13A2 RUVBL2 LIG3 KCNQ3 PCNT RALGPS1 ZER1 CCM2 CACNA1A

CACNA1D DSCAML1 ADCY1 DAZAP1 PKD2L2 SNAPC4

11 common elements in "Rabin downreg", "Krach downreg" and "TDP-43 clip": STMN2 ATP8A2

MAP2 ADARB1 MAP3K9 HSPA12A UBE2O ELAVL3 WDR63 UBE3C SLC8A3

134 common elements in Krach downreg and "TDP-43 clip": ANO3 CUX2 RNFT2 MADD

DNAJC25 KIAA1841 CRB1 TNS1 USP13 MAP2K4 XYLB UCK2 DISP1

MMP16 TAPT1 SIL1 C1orf216 CAMK1D IFT122 STX1B EPB41 MAPK8IP3

VPS13A HTT PRKRIP1 TUBA1A IPO11 PDK3 DGKI HECW2 CHRM2 RNF157

POLR1A RCC2 MYCBP2 LRP8 PSD3 ANKRD50 GABRA3 GNB5 DLGAP1

SARS ENTPD4 FOCAD MAPK10 PFKP HERC1 TLN2 NBAS PSME4 SLIT2 TUBB

OSBPL9 PEG10 FAM13B REPS2 TMEM178B RUSC1 TBC1D9 ZNF273 SV2C

FUNDC2 CLASP2 SYN2 TUBA1B IRGQ TTBK2 HK1 CLASP1 BDP1 GRIA2

DENND5B ANK2 PITPNA EXOC6B TM7SF3 POLR3A YBX1 STK38 RPL11 PFKFB2

SLC8A1 ZKSCAN8 PALD1 CNOT6L CTSC KIF1C CELF2 MYH9 PHC3 SETD7 RPS8

MIER3 GPT2 VEZF1 STMN1 AGO3 PTPRF ARHGAP26 TRIM8 HNRNPF PTPRA

ZMIZ1 BBX DMD EPC1 INTS3 PRSS23 RNF38 FOXN3 PIP4K2A PABPC1 RPL27A

PTP4A2 TRMT11 RPL35 HP1BP3 SEC11A CTNNB1 RPS6KA2 TIMP2 RPL22

C7orf55-LUC7L2 SPATS2L ARHGAP12 SRSF1 CNTLN AK2 ADAM10

RPL5 ZNF226 HERPUD1 ANXA5 PEA15

Suppl. Table 8. Common up-regulated RNAs

Tam et al 2019	Table S3A	<u>A) List of genic targets bound by TDP-43 eCLIP</u>

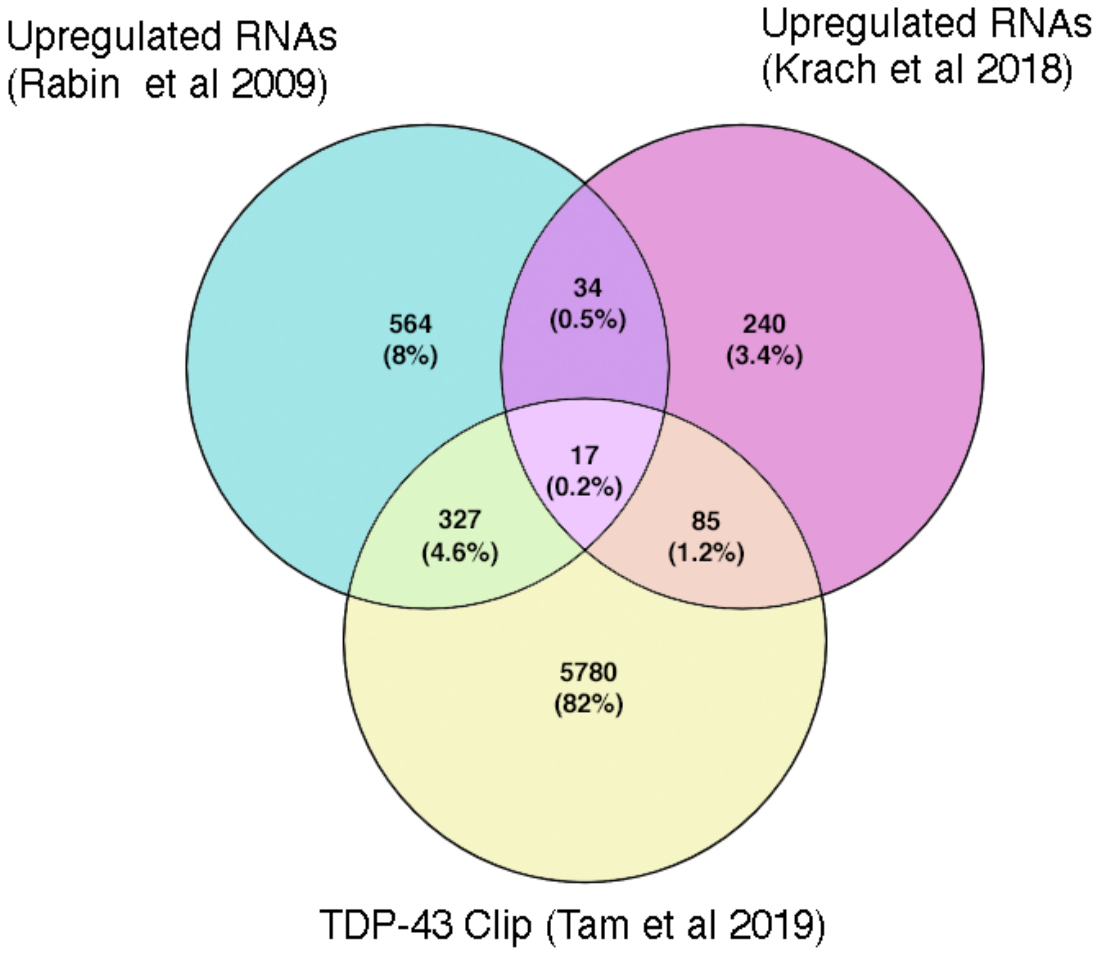

34 common elements in "Rabin upreg" and "Krach upreg":

MAOB	SORBS1	GLIS3	ASAH1	GPNMB	NPC2	IQGAP1	SSFA2	AGT	TWSG1	CYP4F11		
	IL13RA1		C1S	KAL1	NQO1	AHCYL1	RAB31	MCL1	GM2A	CHI3L1	PARP4	NFKBIA
	RCN1	CCDC50	ADAMTS1		ARHGEF6		ITM2C	THBS2	TNFAIP1		CLK4	
	ERBB2IP		VAT1	RPS25	NOL8							

327 common elements in "Rabin upreg"						and "TDP-43 clip":			TSHZ2	AQP1	FNDC3B	
	OGFRL1	ERCC4	LTBP1	IGF2R	SP1	FGF2	ANTXR2		KATNAL1		ABCA1	DDR2
	MAPKAPK5		TNC	CLIC4	PPM1D	DLG5	PLEKHA5		CNOT1	RFX4	BBOX1	SFT2D1
	NUP35	BAZ1A	TMEM65		DNAJC1	DDAH1	SNX7	NTRK3	STK4	ATF7	PIK3C2A	
	SCMH1	SLC16A9		CHUK	MAP3K5		SMARCA1		UTRN	DLST	C7	NUMB
	SMAD4	LASP1	NCOR1	COG5	FUT10	MCCC2	MED13L		VIM	ZMYM1	CD109	SBF2
	KLHL23	ZFX	SDCBP	ANKFY1	TEX2	PROS1	CRLS1	BNC2	CKLF	PRDX6	NCOA1	DAAM1
	ABLIM1	LMAN1	SEC24D	PPFIA1	ANTXR1		TOR1AIP1		USP53	STK38L	TBC1D1	ZEB1

THOC2 SF3B3 RFX3 RPL30 MPZL1 LGR4 SRGAP1 OSBPL11 ABI1 BAMBI TPST1
FNBP1L FARP1 PARD3 GNAI3 PSAP DPYSL3 PTPRZ1 RBM4 PPP2CB CNTN1 GPM6A METTL9
PRKDC PHF20 FBXL7 DYNLT1 ELF1 VCL ITPR2 UBE2E1 GTF2E1 GIT2 PPP1R10
STAG2 ZC3HAV1 DIAPH2 HIF1A POR PRPF4B MTMR3 TXNDC11 RUFY1
PRPF40A CTSB NEBL USP6NL SCYL2 HS2ST1 ST6GALNAC3 LCORL SMARCB1
STAG1 FADS2 FMN2 PPP4R1 CLIP1 ABR ZFAND3 PDPR PLCE1 ZRANB1
NIN BFAR HNRNPA1 REST APPBP2 ARFIP1 LSM14A ST5 CORO1C
ASCC1 CTCF DAD1 TEAD1 BMPR1B PDIA6 RAB5C CNN3 PARD3B ELF2
DTNA USP3 USP47 ATF6 RASSF8 CP STAU1 ITM2B PIGG CROT SS18 OPHN1
GOLM1 TBL1X MANBA ARNT USP28 SFT2D2 CFH CCDC47 LRP1 YME1L1 CASP8AP2
CLU ATP2B4 PDPN BMPR1A RBBP8 CLNS1A H3F3B PHIP BRWD3 UBE2R2 NEO1
ADAM17 DDB2 CFDP1 MAPKAP1 HSP90B1 PPP2R5C ASPH YWHAZ
RHPN2 ID3 REEP3 VCAN SLC16A2 COMT DUSP16 EIF4E2 ARHGEF3 YAP1
SMC1A DENND4C AZIN1 RER1 RDX GLG1 CNOT6 TMED5 ACTN1 IQGAP2 DKC1
MINPP1 GPC6 HEXA DPYD TTC19 RUVBL1 PNPLA8 CRNKL1 NFE2L2 SOD2
ACVR2A FN1 LAMB1 RBL2 FYN SMAD9 CLK1 APPL2 ADAMTS9 SERBP1
CGGBP1 ACOX1 CALU HSDL2 SLC23A2 RPN2 TNKS2 CCT3 STK3 HIPK1
POLA1 SRBD1 RGS10 SMU1 EDNRA RAB28 TLE4 CPE ZZZ3 LSM6 ADD3 NFXL1
MTPN SETD5 UNC13B TANK GRN SLC35F5 PCGF5 NEK1 SLC20A2
TUBB PPP1CB ASCC3 BMI1 IRF2 TBCD PFKFB3 CHD2 RSF1 SLC39A11 TUG1
YES1 FUBP1 HEATR1 CUL4A CAPN2 MEX3C EP300 WASF2 GSTP1 TNPO1 SEC31A
CARHSP1 CAPRIN1 RPS29 CDC14B SLC39A14 ARHGEF12 ITGAV RAP1B
MGP AATF PDE7A CST3 SMCHD1 RASA2 RBBP6 MAP3K2 GRINA PLXDC2
NSL1 THRB SDC2 MRPL3 AP2B1 RAB23 FZD6 SEL1L P4HA1 PTK2 ARHGEF10
EIF3H MMP2 DCAKD MLLT10 CPSF2 SCAMP2

17 common elements in "Rabin upreg", "Krach upreg" and "TDP-43 clip": PHKA1 PRKCA
HERPUD1 CTNNA1 PALLD PRKD3 CYFIP1 LAMC1 LUZP2 B2M ANXA5 RNF38
MYH9 HP1BP3 CTNNB1 ADAM10 TIMP2

85 common elements in "Krach upreg" and "TDP-43 clip": CDKN1A LPCAT2 FRMD5 ETS1
ELK4 SLC6A6 NLRP1 GCH1 NEDD9 HMGB2 PHF10 LIMA1 RPS6 FAT4 FILIP1 MEF2C
TCF12 PER3 SLC6A8 CSNK1E RGS5 PHF21A PROX1 KLHL5 NPAS3 VGF SYT17 RPL36
CREB3L2 SNX5 RPS4X FLNB RALGDS TIMP3 AK4 RPS12 YBX1 STK38 RPL11
PFKFB2 SLC8A1 ZKSCAN8 PALD1 CNOT6L CTSC KIF1C CELF2 PHC3 SETD7 RPS8
MIER3 GPT2 VEZF1 STMN1 AGO3 PTPRF ARHGAP26 TRIM8 HNRNPF PTPRA
ZMIZ1 BBX DMD EPC1 INTS3 PRSS23 FOXN3 PIP4K2A PABPC1 RPL27A PTP4A2
TRMT11 RPL35 SEC11A RPS6KA2 RPL22 C7orf55-LUC7L2 SPATS2L
ARHGAP12 SRSF1 CNTLN AK2 RPL5 ZNF226 PEA15

Suppl. Table 9. Predicted Functional partners of ELAVL3

https://string-db.org/network/9606.ENSP00000352162

**Predicted Functional Partners:**			
	LGALS4	*Galectin-4; Galectin that binds lactose and a related range of sugars. May be involved in the assembly of adherens junctions; Galectins*	0.923
	GFAP	*Glial fibrillary acidic protein; GFAP, a class-III intermediate filament, is a cell- specific marker that, during the development of the central nervous system, distinguishes astrocytes from other glial cells*	0.843
	NEUROG1	*Neurogenin-1; Acts as a transcriptional regulator. Involved in the initiation of neuronal differentiation. Activates transcription by binding to the E box (5'-CANNTG-3'). Associates with chromatin to enhancer regulatory elements in genes encoding key transcriptional regulators of neurogenesis (By similarity); Basic helix-loop-helix proteins*	0.782
	RBFOX3	*RNA binding protein fox-1 homolog 3; Pre-mRNA alternative splicing regulator. Regulates alternative splicing of RBFOX2 to enhance the production of mRNA species that are targeted for nonsense-mediated decay (NMD); RNA binding motif containing*	0.737
	DNAJC2	*DnaJ homolog subfamily C member 2; Acts both as a chaperone in the cytosol and as a chromatin regulator in the nucleus. When cytosolic, acts as a molecular chaperone: component of the ribosome-associated complex (RAC), a complex involved in folding or maintaining nascent polypeptides in a folding-competent state. In the RAC complex, stimulates the ATPase activity of the ribosome-associated pool of Hsp70-type chaperones HSPA14 that bind to the nascent polypeptide chain. When nuclear, mediates the switching from polycomb- repressed genes to an active state: specifically recruited at hist [...]*	0.725
	L3MBTL1	*Lethal(3)malignant brain tumor-like protein 1; Polycomb group (PcG) protein that specifically recognizes and binds mono- and dimethyllysine residues on target proteins, therey acting as a 'reader' of a network of post- translational modifications. PcG proteins maintain the transcriptionally repressive state of genes: acts as a chromatin compaction factor by recognizing and binding mono- and dimethylated histone H1b/HIST1H1E at 'Lys-26' (H1bK26me1 and H1bK26me2) and histone H4 at 'Lys-20' (H4K20me1 and H4K20me2), leading to condense chromatin and repress transcription. Recognizes and bi [...]*	0.712
	ZPR1	*Zinc finger protein ZPR1; Acts as a signaling molecule that communicates proliferative growth signals from the cytoplasm to the nucleus. Plays a role for the localization and accumulation of the survival motor neuron protein SMN1 in sub-nuclear bodies, including gems and Cajal bodies. Induces neuron differentiation and stimulates axonal growth and formation of growth cone in spinal cord motor neurons. Plays a role in the splicing of cellular pre-mRNAs. May be involved in H(2)O(2)-induced neuronal cell death; Belongs to the ZPR1 family*	0.695
	NEUROD1	*Neurogenic differentiation factor 1; Acts as a transcriptional activator: mediates transcriptional activation by binding to E box-containing promoter consensus core sequences 5'-CANNTG-3'. Associates with the p300/CBP transcription coactivator complex to stimulate transcription of the secretin gene as well as the gene encoding the cyclin-dependent kinase inhibitor CDKN1A. Contributes to the regulation of several cell differentiation pathways, like those that promote the formation of early retinal ganglion cells, inner ear sensory neurons, granule cells forming either the cerebellum or [...]*	0.669
	RBFOX1	*RNA binding protein fox-1 homolog 1; RNA-binding protein that regulates alternative splicing events by binding to 5'-UGCAUGU-3' elements. Regulates alternative splicing of tissue-specific exons and of differentially spliced exons during erythropoiesis*	0.662
	GAP43	*Neuromodulin; This protein is associated with nerve growth. It is a major component of the motile "growth cones" that form the tips of elongating axons. Plays a role in axonal and dendritic filopodia induction*	0.659

Your Current Organism:

Homo sapiens

NCBI taxonomy Id: 9606

Other names: H. sapiens, Homo sapiens, human, man

Interactors https://thebiogrid.org/108310

Interactor	Role	Organism	Experimental Evidence Code	Dataset	Throughput	HTP Score	Curated By	More
A2M	BAIT	**H. sapiens**	**Two-hybrid**	Soler-Lopez M (2011)	**Low**	-	BioGRID	-
CDC37	BAIT	**H. sapiens**	**Two-hybrid**	Soler-Lopez M (2011)	**Low**	-	BioGRID	-
ECSIT	BAIT	**H. sapiens**	**Two-hybrid**	Soler-Lopez M (2011)	**Low**	-	BioGRID	-
ELAVL2	BAIT	**H. sapiens**	**Affinity Capture-MS**	Huttlin EL (2017)	**High**	1	BioGRID	
EWSR1	HIT	**H. sapiens**	**Two-hybrid**	Rual JF (2005)	**High**	-	BioGRID	-
MAST1	BAIT	**H. sapiens**	**Two-hybrid**	Soler-Lopez M (2011)	**Low**	-	BioGRID	-
NOS3	BAIT	**H. sapiens**	**Two-hybrid**	Soler-Lopez M (2011)	**Low**	-	BioGRID	-
NTRK1	BAIT	**H. sapiens**	**Affinity Capture-MS**	Emdal KB (2015)	**High**	-	BioGRID	-
PEX14	HIT	**H. sapiens**	**Co-fractionation**	Wan C (2015)	**High**	0.0763	BioGRID	
RBM39	BAIT	**H. sapiens**	**Affinity Capture-MS**	Han T (2017)	**High**	-	BioGRID	
RNF32	BAIT	**H. sapiens**	**Two-hybrid**	Soler-Lopez M (2011)	**Low**	-	BioGRID	-
SRPK2	BAIT	**H. sapiens**	**Biochemical Activity**	Lipp JJ (2015)	**High**	-	BioGRID	

Suppl. Table 10. Phosphosites

https://www.phosphosite.org/proteinAction.action?id=1299918&showAllSites=true

Residue	ELAVL3 (human)	LTP	HTP	PTM
K69	IGDIESCKLVRDKIT	0	1	ub
K95	SDPNDADKAINTLNG	0	1	ub
K104	INTLNGLKLQTKTIK	0	1	ub
S119-p	VSYARPSsASIRDAN	0	2	phospho
K134	LYVSGLPKTMsQKEM	0	1	ub
S137-p	SGLPKTMsQKEMEQL	0	1	ub
K139	LPKTMsQKEMEQLFS	0	1	phospho
K209	FANNPSQKTGQALLT	0	2	ub
R225-m1	LYQSSARryAGPLHH	0	4	methilation
Y226-p	YQSSARryAGPLHHQ	0	2	phospho
T234-p	AGPLHHQtQRFRLDN	0	1	phospho
R236	PLHHQtQRFRLDNLL	0	3	phospho
R236	PLHHQtQRFRLDNLL	0	2	ub
S251	NMAYGVKSPLSLIAR	0	2	ub
T331-p	CKGFGFVtMTNYDEA	0	1	phospho
S358-p	GERVLQVsFKTSKQH	0	1	phospho

Supplementary Acknowledgment

The dataset(s) used for the analyses described in this manuscript were obtained from the Age-Related Eye Disease Study (AREDS) Database found at https://www.nei.nih.gov/research/clinical-trials/age-related-eye-disease-study-areds through dbGaP accession number phs000001.v3.p1. Funding support for AREDS was provided by the National Eye Institute (N01-EY-0-2127). We would like to thank the AREDS participants and the AREDS Research Group for their valuable contribution to this research. The Framingham Heart Study is conducted and supported by the National Heart, Lung, and Blood Institute (NHLBI) in collaboration with Boston University (Contract No. N01-HC-25195 and HHSN268201500001I). This manuscript was not prepared in collaboration with investigators of the Framingham Heart Study and does not necessarily reflect the opinions or views of the Framingham Heart Study, Boston University, or NHLBI. Funding to support the Omni cohort recruitment, retention and examination was provided by NHLBI Contract N01-HC-25195 and HHSN268201500001I, as well as NHLBI grants R01-HL070100, R01-HL076784, R01-HL-49869, and U01-HL-053941. Research support to collect data and develop an application to support this project was provided by 3P50CA093459, 5P50CA097007, 5R01ES011740, and 5R01CA133996. The WHI program is funded by the National Heart, Lung, and Blood Institute, National Institutes of Health, U.S. Department of Health and Human Services through contracts HHSN268201600018C, HHSN268201600001C, HHSN268201600002C, HHSN268201600003C, and HHSN268201600004C. This manuscript was not prepared in collaboration with investigators of the WHI, has not been reviewed and/or approved by the Women's Health Initiative (WHI), and does not necessarily reflect the opinions of the WHI investigators or the NHLBI. Funding support for WHI GARNET was provided through the NHGRI Genomics and Randomized Trials Network (GARNET) (Grant Number U01 HG005152). Assistance with phenotype harmonization and genotype cleaning, as well as with general study coordination, was provided by the GARNET Coordinating Center (U01 HG005157). Assistance with data cleaning was provided by the National Center for Biotechnology Information. Funding support for genotyping, which was performed at the Broad Institute of MIT and Harvard, was provided by the NIH Genes, Environment and Health Initiative [GEI] (U01 HG004424). The datasets used for the analyses described in this manuscript were obtained from dbGaP at http://www.ncbi.nlm.nih.gov/sites/entrez?db=gap through dbGaP accession numbers phs000001, phs000007, phs000101, phs000187, phs000196, phs000248, phs000292, phs000304, phs000315, phs000368, phs000394, phs000397, phs000404, phs000421, phs000428, phs000454, phs000615, phs000675, phs000801, and phs000869. The authors acknowledge the contribution of data from Hepatitis C Pathogenesis and the Human Genome supported by 1X01HG005271-01 and R01DA013324 and accessed through dbGaP to the analysis presented in this publication. Funding support for the Genes and Blood Clotting Study was provided through the NIH/NHLBI (R37 HL039693). The Genes and Blood Clotting Study is one of the Phase 3 studies as part of the Gene Environment Association Studies (GENEVA) under GEI. Assistance with genotype cleaning was provided by the GENEVA Coordinating Center (U01 HG004446). Funding support for DNA extraction and genotyping, which was performed at the Broad Institute, was provided by NIH/NHLBI (R37 HL039693). Additional support was provided by the Howard Hughes Medical Institute. The dataset(s) used for the analyses described in this manuscript were obtained from the database of Genotype and Phenotype (dbGaP) found at http://www.ncbi.nlm.nih.gov/gap through dbGaP accession number phs000368. Samples and associated phenotype data for the Genome-Wide Association Scan [GWAS] of Polycystic Ovary Syndrome Phenotypes were provided by Andrea Dunaif, M.D. The authors acknowledge the contribution of data from Genetic Architecture of Smoking and Smoking Cessation accessed through dbGaP. Funding support

for genotyping, which was performed at the Center for Inherited Disease Research (CIDR), was provided by 1 X01 HG005274-01. CIDR is fully funded through a federal contract from the National Institutes of Health to The Johns Hopkins University, contract number HHSN268200782096C. Assistance with genotype cleaning, as well as with general study coordination, was provided by the Gene Environment Association Studies (GENEVA) Coordinating Center (U01 HG004446). Funding support for collection of datasets and samples was provided by the Collaborative Genetic Study of Nicotine Dependence (COGEND; P01 CA089392) and the University of Wisconsin Transdisciplinary Tobacco Use Research Center (P50 DA019706, P50 CA084724). The dataset(s) used for the analyses described in this manuscript were obtained from the Genetics of Fuchs' Endothelial Corneal Dystrophy (FECD) Study through dbGaP accession number phs000421. The grants that have funded the enrollment of the cases and controls to be used in this GWAS are: R01EY016514 (DUEC, PI: Gordon Klintworth), R01EY016482 (CWRU, PI: Sudha Iyengar), and 1X01HG006619-01 (PI: Sudha Iyengar, Natalie Afshari). We would like to thank the FECD participants and the FECD Research Group for their valuable contribution to this research. The authors acknowledge the contribution of data from CIDR-NIDA Study of HIV Host Genetics accessed through dbGaP. Funding support for genotyping, which was performed at the Center for Inherited Disease Research (CIDR), was provided by 1 X01 HG005275-01A1. Funding support for collection of datasets and samples was provided by NIDA grants R01DA026141(Johnson); R01DA004212 (Watters); U01DA006908 (Watters); R01DA009532 (Bluthenthal); as well as the San Francisco Department of Public Health; SAMHSA; and HRSA. The Genome-Wide Association Study (GWAS) of Non-Hodgkin Lymphoma (NHL) project was supported by the intramural program of the Division of Cancer Epidemiology and Genetics (DCEG), National Cancer Institute (NCI), National Institutes of Health (NIH). The datasets have been accessed through the NIH database for Genotypes and Phenotypes (dbGaP) under accession # phs000801. A full list of acknowledgements can be found in the supplementary note (Berndt SI et al., Nature Genet., 2013, PMID: 23770605). This study made use of data generated by investigators in the BEACON consortium through a grant funded by the US National Institutes of Health (NIH) (RO1CA136725) to Thomas L. Vaughan and David C. Whiteman (multiple PIs). In support of this work, T.L.V. was also supported by NIH grant KO5CA124911 and D.C.W. by a Future Fellowship grant FT0990987 from the Australia Research Council. RHR is supported through philanthropic support from Dr. Peter Buck. Additional collaborators, sources of support and origin of the data and biospecimens are listed in the following publication: Levine DM, Ek WE, Zhang R, Liu X, Onstad L, Sather C, et al. A genome-wide association study identifies new susceptibility loci for esophageal adenocarcinoma and Barrett's esophagus. Nat Genet. 2013 Dec;45(12):1487–93. This work was supported in part by the Intramural Research Programs of the NIH, National Institute on Aging (Z01-AG000949-02). This study used the high-performance computational capabilities of the Biowulf Linux cluster at the National Institutes of Health, Bethesda, Maryland, USA (http://biowulf.nih.gov). We thank the Laboratory of Neurogenetics (NIH) staff for their collegial support and technical assistance.
